# Electrically Conductive Amine Functionalized Reduced Graphite Oxide Foam for CO_2_ Removal from the Air

**DOI:** 10.1021/acsami.5c15229

**Published:** 2025-11-18

**Authors:** MinGyu Song, Jaedeok Kim, Christopher W. Jones, Ryan P. Lively

**Affiliations:** School of Chemical & Biomolecular Engineering, 1372 Georgia Institute of Technology, Atlanta, Georgia 30332, United States

**Keywords:** direct air capture, Joule heating, vacuum swing adsorption, graphene aerogel, amines

## Abstract

Rapid regeneration of CO_2_ adsorbents is critical to improving the productivity of direct air capture (DAC) systems. In this study, we codesigned a material to have appropriate electrical conductivity and CO_2_ adsorption properties to enable efficient CO_2_ capture from air. Specifically, we present a poly­(ethylenimine) (PEI)-impregnated thermally annealed graphite oxide (TAGO900) foam adsorbent tailored for vacuum-assisted electrically driven thermal swing adsorption (V-ETSA). This structured adsorbent leverages the high electrical conductivity of the reduced graphite oxide framework to enable fast and direct heating of the adsorbent material by electrical resistance heating (Joule heating). An optimal sample, 40 wt % PEI (molecular weight 25k) impregnated TAGO900, shows the best balance between adsorption capacity (1.54 mmol g^–1^) and adsorption rates (0.016 mmol g^–1^ min^–1^) using fixed bed breakthrough experiments at 25 °C and 70% RH using 50 sccm 400 ppm of CO_2_/N_2_ flow. Compared to conventional temperature vacuum swing adsorption (TVSA), the V-ETSA approach achieves substantially faster CO_2_ desorption, achieving average desorption rates (including cooling time) of 0.09 mmol g^–1^ min^–1^—approximately 2.5 times faster than TVSA under similar operating conditions. The maximum desorption rate reaches 0.23 mmol/g/min during the desorption stage. These results underscore the importance of the direct heating strategy, such as Joule heating, for fast and highly productive vacuum swing adsorption in DAC systems.

## Graphical abstract

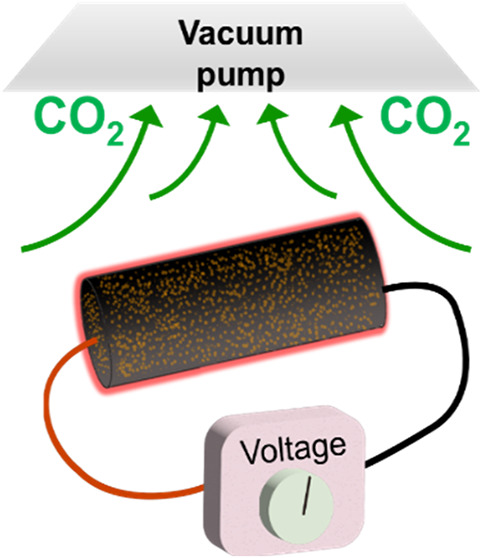

## 1 Introduction

Along with efforts to mitigate CO_2_ emissions and global warming, separating CO_2_ from different sources and storing the CO2 permanently is considered one of the most promising ways of addressing anthropogenic CO_2_ emissions.^1^ CO_2_ capture technologies can be classified into “avoided emissions” and “negative emissions,” depending on whether they target emitted CO_2_ sources (e.g., power plants) or ambient air.^2^ These categories are considered reciprocal, as one reduces current anthropogenic CO_2_ emissions while the other removes historical CO_2_ accumulation.^2,3^ Direct air capture (DAC), a technology that removes CO_2_ directly from the atmosphere, is increasingly recognized as a contributor to the needed array of solutions to rising CO_2_ emissions.^2,4^ Among various DAC methods, adsorption-based approaches are emerging as scalable and potentially cost-effective options, evidenced by the fact that 65% of existing DAC companies use solid adsorbents as their capture medium.^5^

In 1999, Lackner et al. proposed the concept of DAC for climate stabilization. A decade later, since 2009, porous materials functionalized by amines (solid-supported amines) have been one of the most well-studied DAC material classes, with an initial focus on their adsorption capacity.^2,6^ For example, the Jones group reported solid-supported amines (hyperbranched aminosilica) for DAC application in 2009.^7,8^ In parallel, the Sayari group demonstrated amine-grafted MCM-41 for removing dilute concentration (400 ppm) CO_2_, resulting in 0.98 mmol of captured CO_2_ per gram of adsorbent at 25 °C under dry air.^9^ Jones and co-workers reported various solid supports impregnated with grafted or physically impregnated polymeric amines, studying the influence of the support materials on adsorption performance and the behavior of different amines.^10–14^ Among more recent approaches, Long utilized diamine functionalized Mg_2_(olz) metal organic frameworks (MOFs) to overcome the low carbon capture efficiency of the diamine appended Mg_2_(dobpdc) under ultradilute DAC conditions, a limitation previously reported by Darunte et al.^15,16^ Yaghi and his team published a polymeric amine grafted covalent organic framework (COF) for DAC with high CO_2_ capture capacity (2.05 mmol g^–1^) under humid DAC conditions (50% RH and 25 °C), successfully expanding the library of DAC materials that operate under ideal desorption conditions.^17^ Unfortunately, most academic studies of DAC, including those discussed above, use inert gas as a desorption carrier gas, producing a nonconcentrated CO_2_ product.

The use of inert gas purge has numerous advantages, such as isolation of variables related to desorption (sorbents degradation and fractional desorption and sorbent reactivation), a facile desorption setup, and straightforward monitoring of desorbed gases, beneficial for an early material development stage. However, the dearth of studies of developed materials under practical desorption conditions raises uncertainty about the effective removal of accumulated CO_2_ from the sorbents, slowing the pace of meeting the CO_2_ removal targets (85 and 980 Mt of CO_2_ in 2030 and 2050) proposed by the International Energy Agency (IEA).^18^

Among the various practical desorption strategies, direct steam desorption (including steam assisted temperature vacuum swing adsorption, S-TVSA) is one of the most efficient and rapid methods for CO_2_ removal, primarily due to the swift heat transfer provided by steam, first proposed for amine-based DAC adsorbents by Li and co-workers.^19–21^ However, challenges such as uncontrolled loss of water, leaching or agglomeration of active CO_2_ adsorption components (like amines), and metal contamination from low-purity steam can lead to the deactivation of the adsorbent.^20–27^ Furthermore, when the adsorption temperature drops below zero degrees Celsius, residual water from the steam can freeze, obstructing the adsorption column. In this context, temperature vacuum swing adsorption (TVSA) presents an appealing alternative. This method operates by creating a temperature and pressure difference to desorb adsorbates, eliminating contact with a liquid medium. Nevertheless, the slow heat transfer that occurs during TVSA using indirect heating often results in longer desorption times compared to direct steam desorption, thereby reducing overall productivity.^20^ For instance, the thermal conductivity of fumed silica (pore diameter ∼ 100 nm) decreased by 73% at 0.1 bar compared to 1 bar air pressure due to the reduced heat conduction by gas molecules.^28^ Indeed, Wurzbacher et al. reported a slower temperature ramping under lower pressure conditions during TVSA operation after CO_2_ capture from the humid air (25 °C and 40% RH).^29^

While thermal swing adsorption processes have been extensively studied, limited attention has been paid to the design of adsorbent materials that enable direct electrical heating. Bridging this gap requires a fundamentally different approach to materials synthesis—one that integrates electrical conductivity, thermal responsiveness, and affinity to ultra dilute CO_2_ (400 ppm) in a porous, structurally stable framework. In this study, we developed a structured adsorbent specifically designed for vacuum-assisted electrical thermal swing adsorption (V-ETSA) to achieve fast desorption kinetics using a direct and fast-response Joule heating method. The desirable characteristics for an adsorbent suitable for V-ETSA include high CO_2_ uptake, stability throughout the process, low pressure drop, and rapid/efficient heating.^30–32^ With this purpose, we focused on carbonaceous materials, as they are readily available, tunable, porous, and electrically conductive.^33^ Several recent examples suggest carbon materials have promising properties for DAC, including amine/silica/cellulose acetate composite coated carbon fibers studied for V-ETSA, an electrolyte charged activated carbon electrode regenerated by Joule heating, and an imidazole functionalized carbon nanotube for solar heat driven regeneration.^34–37^ Utilizing graphite oxide, we fabricated an adsorbent contactor capable of electric heating through Joule heating by hydrothermal reaction and thermal annealing without additional chemical reducing agents. To enhance the CO_2_ adsorption capacity of the thermally annealed reduced graphite oxide foam (TAGO), 800 or 25k Da branched poly­(ethylenimine) (PEI) were infused into the TAGO by lyophilization. In comparative tests, our V-ETSA process demonstrated superior desorption capacity (0.94 vs 0.6 mmol g^–1^), kinetics (0.091 vs 0.036 mmol g^–1^ min^–1^), CO_2_ purity (77 vs 70%), and a three times lower thermal energy use when compared to use of traditional TVSA methods with the same adsorbent material.

## 2 Results and Discussion

### 2.1 Synthesis of rGO Foam and Electrical Resistance Optimization

Carbon-based materials are particularly suitable for electrically driven thermal swing adsorption (ETSA) because their electrical resistance and porosity can be readily tuned as schematically illustrated in Figure 1.^33,35,37–39^ As a preliminary study toward developing an electrically conductive structured adsorbent, we selected reduced graphite oxide (rGO) powder impregnated with amines. Figure S1 displays the CO_2_ adsorption capacities at 400 ppm for poly­(ethylenimine) (PEI) impregnated rGO with two different molecular weights—800 Da (PEI800) and 25k Da (PEI25k)—measured gravimetrically at 30 °C and 50% relative humidity (RH). Increasing the amine loading enhanced the CO_2_ adsorption capacity in both PEI800 and PEI25k samples due to the greater availability of amine groups for CO_2_ capture. Notably, the higher molecular weight PEI25k exhibited lower adsorption capacities compared to the lower molecular weight PEI800. This decrease can be attributed to the higher viscosity of PEI25k, which increases the diffusion resistance and reduces the availability of active amine sites.^40^ Overall, the significant CO_2_ uptakes (∼3.2 mmol g^–1^ for PEI800 and ∼ 2.2 mmol g^–1^ for PEI25k) demonstrate the potential of GO-based materials as effective amine carriers for direct air capture (DAC) applications.

**Figure 1:**
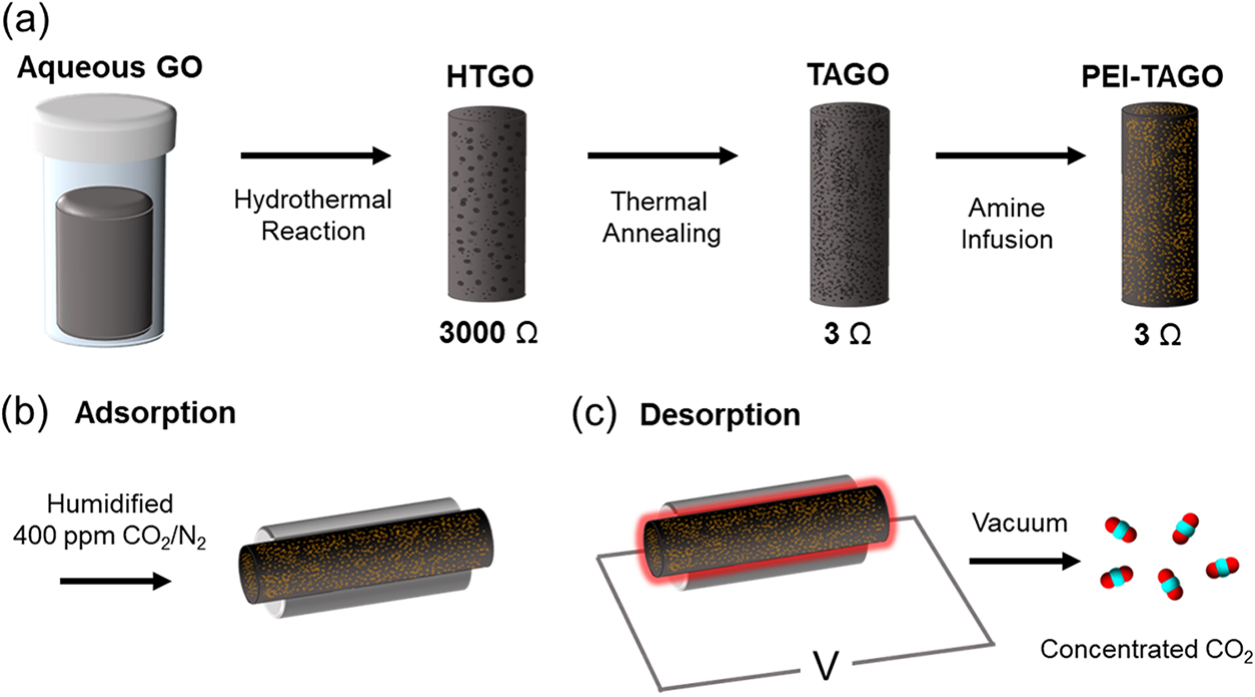
(a) Schematic illustration of the amine functionalized graphite oxide (GO) foam fabrication and vacuum-assisted electrical thermal swing (b) adsorption and (c) desorption process. Black dots in hydrothermally treated GO (HTGO) and thermally treated GO represent pores in HTGO and TAGO foams, repsectively. Yellow colored foam in PEI-TAGO indicates impregnation of amine inside the TAGO pores.

In practical DAC applications, a gas–solid contactor is essential to structure and display the adsorbent material in the air flow field in an efficient way while still allowing for fast heating/desorption/cooling phases of the cycle. To achieve this, we fabricated reduced graphite oxide (rGO) foams using a hydrothermal method followed by lyophilization (as shown in Figures S2 and S3) without chemical reducing agents.^41^ The electrical resistance of the resulting hydrothermally treated graphite oxide (HTGO) foam was approximately 3000 Ω (Figure 2a, labeled as a sample annealed at 0 °C). Due to the high electrical resistance and possible electrical capacitive effects from oxygen-containing functional groups (∼21 wt % oxygen, Figure 2a),^42^ an appropriate Joule heating (i.e., temperature increase in response to voltage input) was not observed. According to the literature, the electronegativity of oxygen functional groups present in graphene oxide impedes electron flow by trapping electrons.^43^ Therefore, additional thermal annealing under an inert atmosphere (99.999% Ar) was conducted to decrease the oxygen content in the HTGO adsorbent. Thermally annealed graphite oxide (TAGO) foams were successfully produced at various annealing temperatures without structural deformation (Figure S3). As demonstrated in Figure 2a, thermal annealing at 500 °C significantly reduced both the oxygen content (estimated as the residual weight fraction from elemental analysis, EA, in Table S1) and the electrical resistance (∼300 Ω). Higher annealing temperatures further decreased the oxygen content and electrical resistance, reaching as low as 4 wt % oxygen and ∼3 Ω at 900 °C. The reduced *d*-spacings observed in the X-ray diffraction (XRD) patterns at higher annealing temperatures (Figure S4 and Table S2) are consistent with lower oxygen content and reduced spacing between the graphene layers.^44^ To minimize the capacitive effects of residual oxygen functionalities, the rGO foam annealed at 900 °C (TAGO900) was selected for further investigation. Figure 2b shows its Joule heating performance. TAGO900 exhibited excellent Joule heating characteristics, achieving temperatures ranging from 25 °C at 0 V up to 120 °C at 2.5 V (0.96 A), demonstrating a nearly linear Ohmic law relationship between voltage and current.

**Figure 2:**
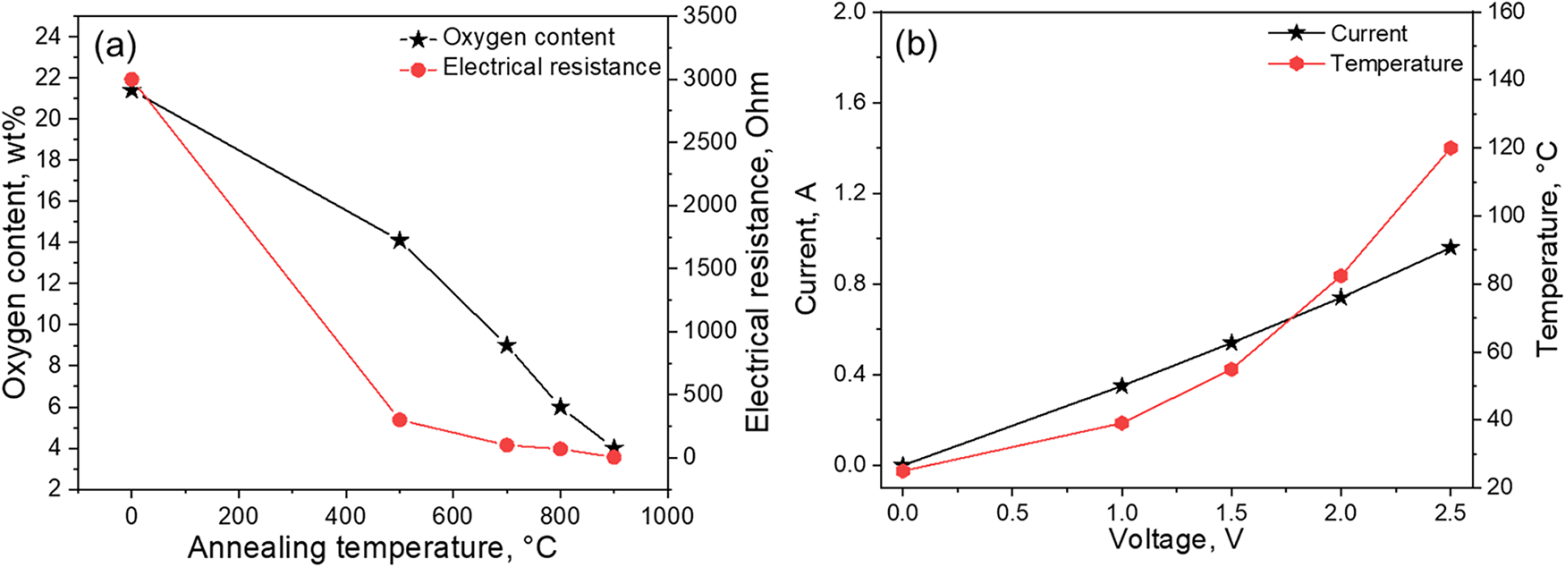
(a) Oxygen content and electrical resistance of TAGO after thermal annealing at different temperatures and (b) the Joule heating properties of TAGO annealed at 900 °C. The sample at 0 °C annealing temperature represents HTGO without thermal annealing. The oxygen content is estimated as the residual weight fraction after elemental analysis (EA). The lnegth of the foam was 1.5 cm.

Thermally annealed TAGO inherently exhibits limited CO_2_ capture capability under direct air capture (DAC) conditions, primarily due to the loss of polar functional groups by thermal annealing. However, preliminary experiments with powdered rGO confirmed promising CO_2_ adsorption capacities upon PEI impregnation (∼3.2 and 2.2 mmol g^–1^ for PEI800 and PEI25k, respectively; Figure S1). Based on these initial results, amine infusion and optimization were performed for TAGO900 foams. To identify the optimal amine loading, aqueous polymeric amine solutions with concentrations ranging from 2.5 to 20 wt % were infused into the foam. Samples were designated based on the amine concentration in the infusion solution and the molecular weight of the PEI. Nitrogen contents of the resulting composite adsorbents, measured by elemental analysis (EA), are summarized in Table S1. For example, infusions using 5 wt % of PEI800 and 2.5 wt % of PEI25k (PEI5(800)-TAGO900 and PEI2.5­(25k)-TAGO900, respectively) resulted in nitrogen contents of 11.3 and 13.1 wt %, respectively, corresponding to approximately 35 and 40 wt % PEI loadings. Although TAGO foams infused with PEI800 showed faster adsorption kinetics and higher CO_2_ capacities compared to PEI25k under similar capture conditions (Figure S5) using a gravimetric adsorption method (TGA), the TAGO900 foam infused with 2.5 wt % of PEI25k (1.66 mmol g^–1^) was selected for further studies due to its superior cycling stability during the vacuum-assisted electrically driven thermal swing adsorption (V-ETSA), as detailed in the subsequent V-ETSA section.

The textural properties of rGO foams were examined following each stage of processing—hydrothermal treatment, thermal annealing, and amine infusion. As shown in Figure 3, scanning electron microscopy (SEM) images of HTGO, TAGO900, PEI2.5­(25k)-TAGO900, and PEI5­(25k)-TAGO900 reveal a characteristic sandwich-like graphene framework with channels on the order of 10^2^ μm. These pores are formed by the self-assembly of rGO layers during the hydrothermal treatment, as the reduction of negatively charged surface functional groups diminishes electrostatic repulsion of rGO layers.^41^ This structural feature facilitates bulk gas convection and diffusion compared to dense structures, enhancing CO_2_ adsorption performance. Interestingly, Figure 3c,3d display a porous polymer network, which may enhance the accessibility of CO_2_ molecules to amine sites. In the literature, the development of porous structures (∼10 μm scale) by lyophilization has been reported by Narayanan et al., who observed such features after cross-linking PEI60k with poly­(ethylene glycol) diglycidyl ether and freeze-drying from an aqueous solution—attributed to the sublimation of ice crystals.^45,46^ On the other hand, PEI5­(25k)-TAGO900 lacked such morphological features, likely due to the formation of a thicker amine film at the higher PEI25k loading, as seen in Figure 3e,3f, which may have disrupted the formation and dispersion of small ice crystals during freeze-drying. On the microscopic scale, SEM images in Figure S6 display the layer-by-layer structure of the rGO foams built by π–π stacking of rGO layers after the thermal treatments, which provides mesopore and micropore porosity to rGO foams. The denser layers found in TAGO900 (Figure S6a) versus HTGO (Figure S6b) suggest a decreased repulsion of surface oxygen groups between rGO layers, supported by a reduced amount of oxygen (Figure 2a and Table S1) and decreased XRD *d*-spacing from 3.55 to 3.37 Å (Figure S4a and Table S2) at higher thermal annealing temperatures. In Raman spectroscopy, two characteristic peaks of graphene-based materials appear at approximately 1350 cm^–1^ (D-band, *I*_d_) and 1600 cm^–1^ (G-band, *I*_g_), with their intensity ratio (*I*_d_/*I*_g_) serving as an indicator of structural disorder.^47^ As shown in Figure S4b, the *I*_d_/*I*_g_ ratio of the TAGO samples decreased with increasing annealing temperature, suggesting the removal of heteroatoms such as oxygen—likely in the form of CO or CO_2_—which introduces structural defects and reduces the crystallite size of the rGO framework.^48^

**Figure 3:**
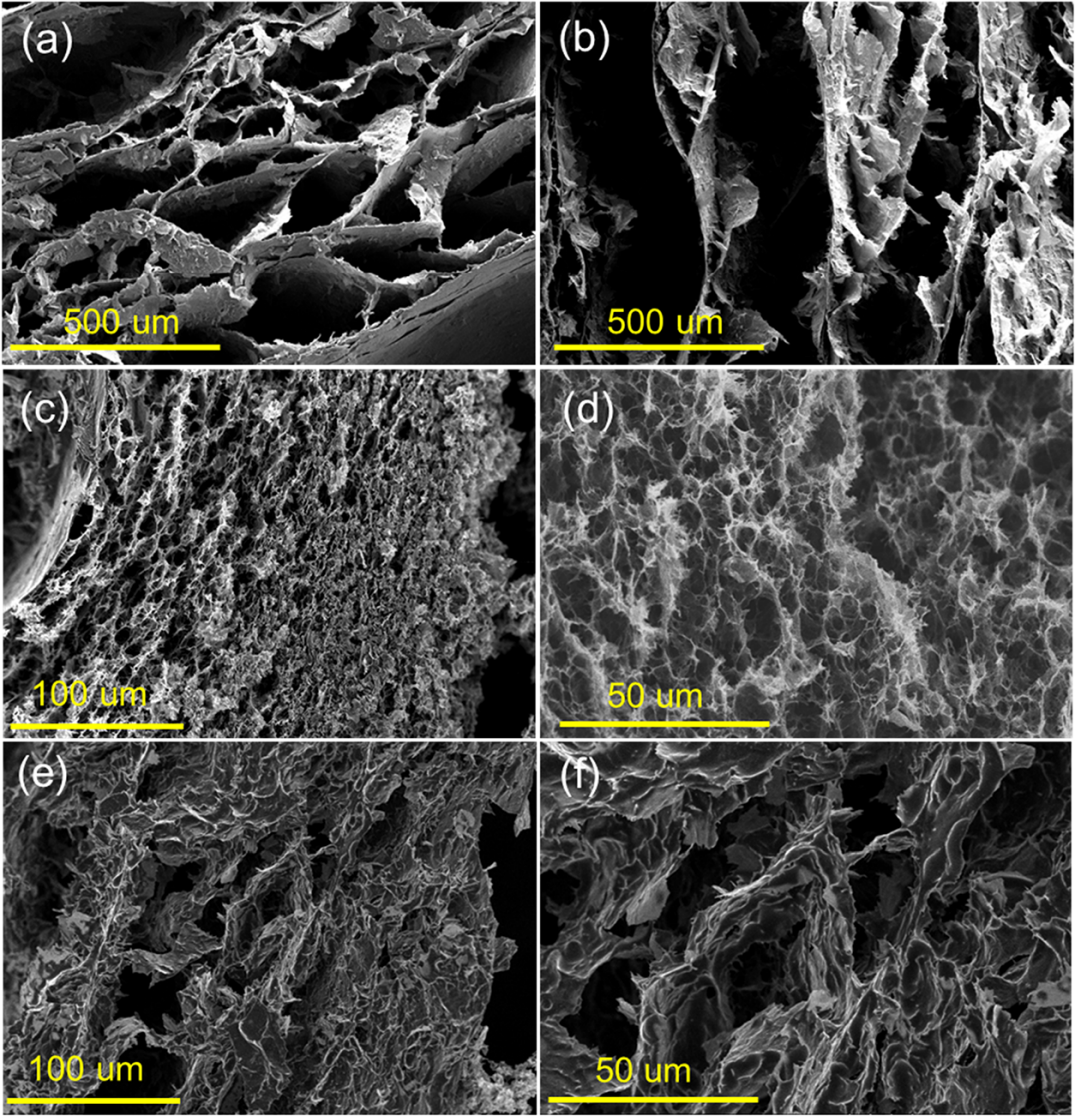
Cross-sectional SEM photographs of (a) HTGO, (b) TAGO900, (c) PEI2.5­(25k)-TAGO900, (d) a magnified version of PEI2.5­(25k)-TAGO900, (e) PEI5­(25k)-TAGO900, and (f) a magnified version of PEI5­(25k)-TAGO900.

In Figure S7, nitrogen physisorption isotherms for HTGO and TAGO900 showcase a type H4 hysteresis loop, indicative of narrow slit-shaped micropores formed during thermal treatment.^49^ Pore size distribution analyses based on N_2_ (Figure S8) and CO_2_ (Figure S9) isotherms, using the quenched solid density functional theory (QSDFT) model tailored for carbonaceous materials with slit-like pores, further confirm the increase in micropore volume following the annealing of HTGO. The loss of microporosity after PEI25k infusion for PEI2.5­(25k)-TAGO900 and PEI5­(25k)-TAGO900 in Figure S8 indicates that the slit-like pores in TAGO900 are blocked by infused amines. Notably, the derivative pore volume of PEI2.5­(25k)-TAGO900 in the mesopore regime increased compared to that of the unmodified TAGO900 and decreased with higher amine loading. Higher BET surface area and pore volume (Table S2) of PEI2.5­(25k)-TAGO900 than PEI5­(25k)-TAGO900, as well as the polymer network found in Figure 3c,d, demonstrate the importance of amine infusion conditions and the effectiveness of lyophilization in developing porosity in PEI25k.

### 2.2 Amine Functionalization of rGO Foam and Its CO_2_ Adsorption Behavior at Different Temperatures and Humidities (−15–40 °C and 20–70% RH)

The CO_2_ adsorption capacities of PEI2.5­(25k)-TAGO900 and PEI5­(25k)-TAGO900 using 400 ppm of CO_2_/N_2_ at various DAC conditions were evaluated using fixed bed breakthrough experiments, as summarized in Figure 4a,4b, respectively. Under dry conditions (0% RH), both samples recorded increased CO_2_ uptake at higher temperatures, indicating that adsorption is primarily limited by diffusional resistance rather than thermodynamics (i.e., lower uptake at lower temperatures). At −15 °C, minimal CO_2_ adsorption was observed for both samples regardless of the humidity, which can be attributed to the restricted mobility of PEI as the adsorption temperature nears its glass transition temperature (−53 °C).^50^ At 5 °C, a strong dependence of CO_2_ uptake on humidity was observed, as the presence of water endows additional mobility to the rigid PEI25k and enables more amines to be accessible to gaseous CO_2_ molecules. Interestingly, at 25 °C, both samples showed the highest adsorption capacities at 50% RH, beyond which the capacities remained constant or slightly declined. This indicates that amine mobility, enhanced by both temperature and water adsorption, reaches an optimal point at ∼50% RH. Additional water beyond this threshold may lead to steric hindrance by pore blockage, limiting CO_2_ access to amine sites.^51^ Similarly, at 40 °C, the relatively small increase in CO_2_ uptake from dry to 20% RH compared to lower temperatures further implies that amine mobility is not limiting under these conditions. Breakthrough CO_2_ adsorption profiles shown in Figure 4c,d for 5 °C and 25 °C, respectively, demonstrate higher or comparable CO_2_ uptake rates at elevated relative humidity for both samples. This further supports the role of water in reducing diffusional resistance by interrupting the hydrogen bonds of amines in PEI25k. The faster adsorption kinetics of PEI2.5­(25k)-TAGO900 compared to PEI5­(25k)-TAGO900 (0.0156 vs 0.009 mmol g^–1^ min^–1^, respectively) are attributed to its more porous structure and presumably thinner amine film, which facilitates CO_2_ mass transfer to the amine domains. Importantly, PEI5­(25k)-TAGO900 required approximately twice the adsorption time to capture an equivalent amount of CO_2_. Such slow kinetics will significantly increase the energy consumption for gas blowers and raise the overall capital cost of the DAC process.^52,53^ Based on this performance comparison, PEI2.5­(25k)-TAGO900 was selected for further investigation.

**Figure 4:**
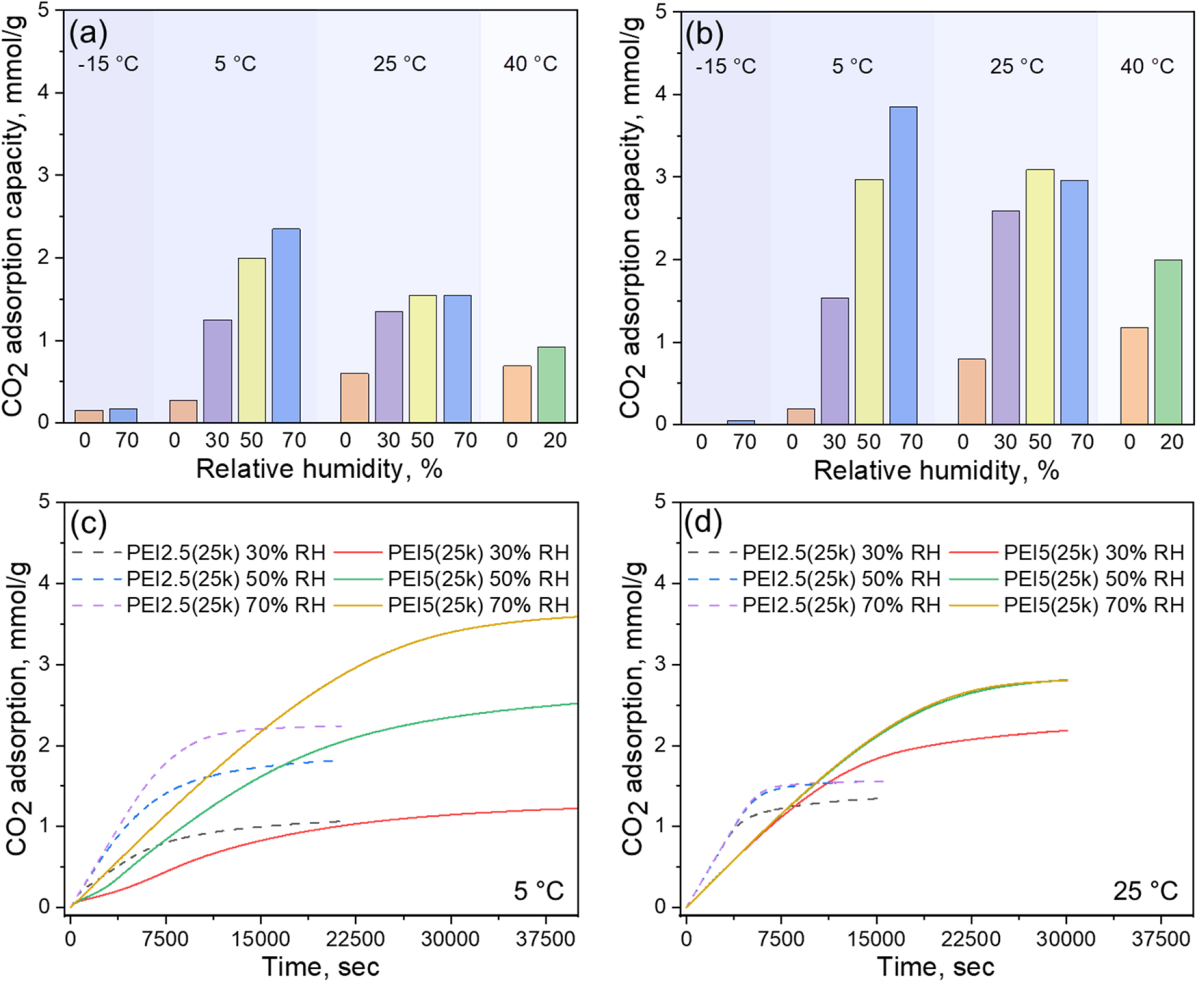
CO_2_ adsorption capacities of (a) PEI2.5­(25k)-TAGO900 and (b) PEI5­(25k)-TAGO900 from fixed bed breakthrough experiments under various DAC conditions and corresponding CO_2_ uptake profiles at (c) 5 °C and (d) 25 °C. The CO_2_ adsorption capacities are based on the pseudoequilibrium capacity (*C*/*C*_0_ = 0.95). Breakthrough profiles showing the outlet CO_2_ concentration are available in Figures S10–S12.

### 2.3 V-ETSA Using Amine-Functionalized rGO Foam

The Joule heating behavior of PEI2.5­(25k)-TAGO900 was evaluated, as presented in Figure 5. Its electrical resistance was independently measured to be approximately 3 Ω using an ohmmeter. This value is comparable to that of the unmodified TAGO900, indicating that the physical incorporation of PEI via the infusion method does not significantly alter the electrical conductivity of the graphitic matrix. Figure 5a shows the current and temperature rise as the input voltage gradually increases. Based on these results, 2.5 V was identified as the optimal voltage, achieving an adsorbent temperature of 120 °C. Subsequently, the heating and cooling cycles at this voltage were examined in Figure 5b,c. The adsorbent was heated to the target temperature within 60 s during the Joule heating process and cooled from 120 °C to 34 °C in approximately 60 s, indicating a total regeneration time of just 120 s. Given that the typical desorption temperature range for PEI-impregnated adsorbents is between 60 and 110 °C,^10–12,54^ the thermal response observed here demonstrates that PEI2.5­(25k)-TAGO900 operates within an effective and practical temperature range for regenerating amine-based adsorbents.

**Figure 5:**
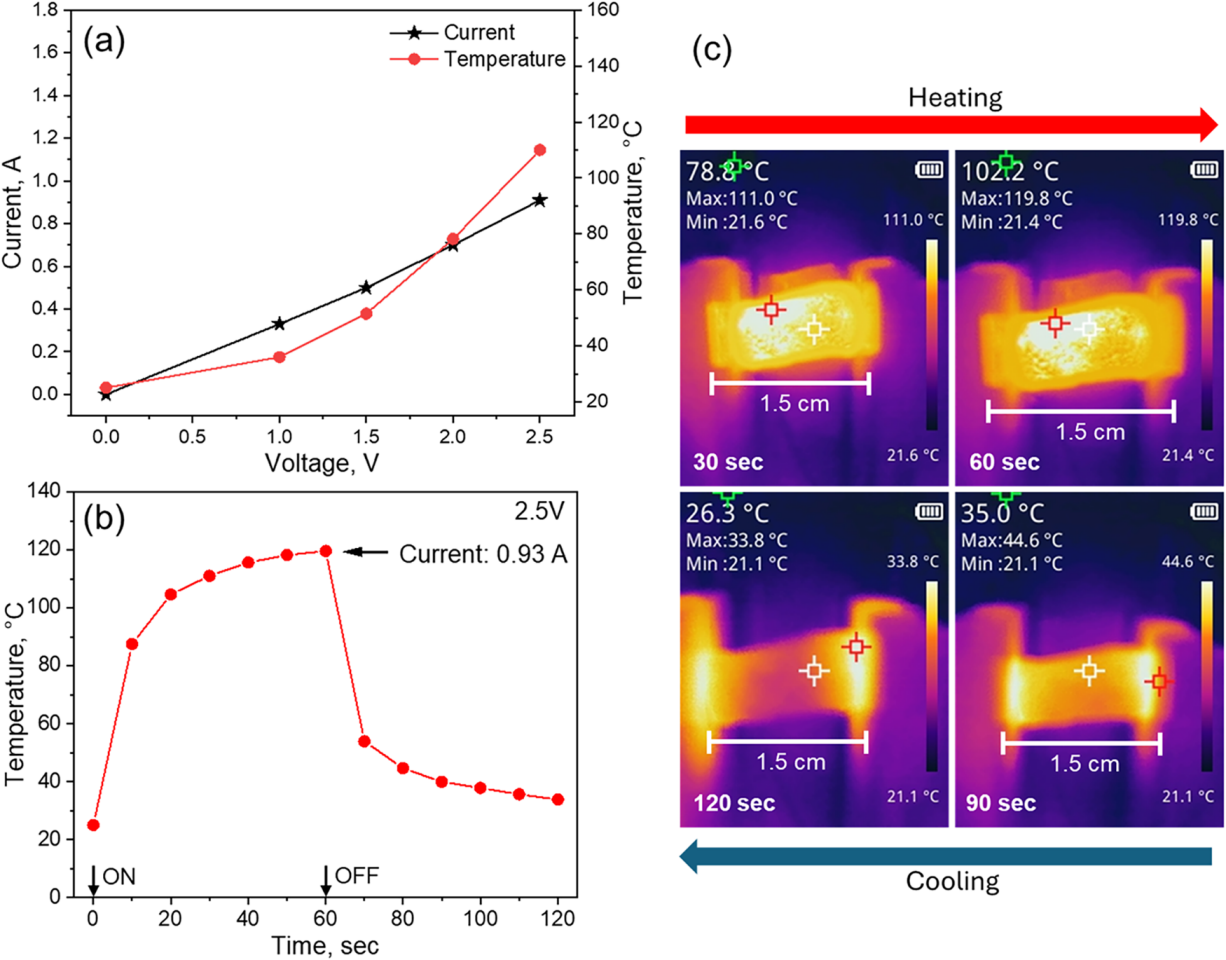
Joule heating properties of a single PEI2.5­(25k)-TAGO900 foam: (a) current and temperature response as a function of input voltage, (b) the temperature profile of the adsorbent during joule heating at 2.5 V (the electrical input was stopped at 60 s), and (c) Joule heating and cooling pictures from an infrared camera. The length of the foam was 1.5 cm.

The desorption performance of the developed Joule heating DAC adsorbent module was evaluated using a customized apparatus (Figure S13) under both temperature swing adsorption (TVSA, with the heat supplied by external heat tape) and V-ETSA conditions, as shown in Figure 6. In the desorption experiments, we used three connected PEI2.5­(25k)-TAGO900 foams using conductive glue to obtain reliable desorption data. A comparison of internal and external temperature profiles highlights inefficient heating of the adsorbent material in the TVSA system (Figure 6a,b), in contrast to V-ETSA (Figure 6c). For TVSA at 90 °C, the outer surface of the module reached 91 °C while the internal temperature only rose to 64 °C at the time heating was stopped (1,100 s). At 120 °C using TVSA, this temperature disparity persisted, with the outer and inner temperatures recorded as 120 °C and 83 °C, respectively. In contrast, during V-ETSA operation, the internal temperature reached 85 °C while the outer temperature remained at 45 °C—demonstrating a significant reduction in parasitic heat loss to the housing material by directly heating the conductive support.

**Figure 6:**
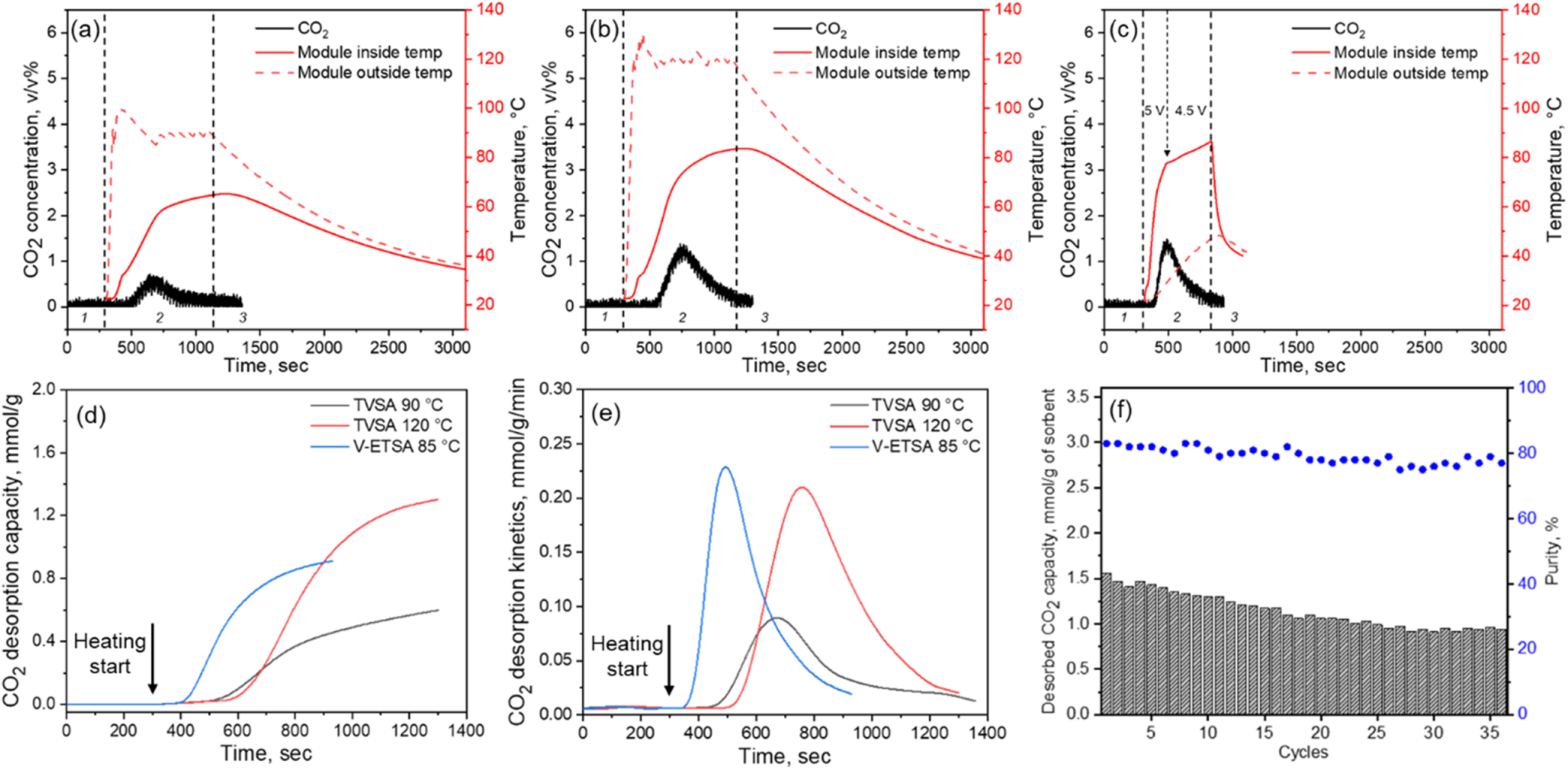
CO_2_ desorption concentration and temperature profiles of the PEI2.5­(25k)-TAGO900 module during (a) TVSA at 90 °C, (b) TVSA at 120 °C, and (c) V-ETSA at 85 °C under vacuum (0.07 bar), following adsorption at 22 °C and 70% RH. For the TVSA conditions, the temperature reflects the set point of the applied external heat source. Corresponding (d) CO_2_ desorption capacity profiles, (e) desorption kinetics, and (f) cyclic CO_2_ desorption capacities and purities are also shown. All desorption processes followed a three-step sequence: (1) vacuum purge, (2) temperature–vacuum desorption, and (3) cooling.

As a result of faster and more efficient heat transfer, the CO_2_ desorption capacity of V-ETSA nearly doubled that of TVSA at a comparable desorption temperature (0.94 vs 0.44 mmol g^–1^ at 900 seconds for V-ETSA and TVSA 90 °C, respectively), and matched the capacity achieved at 900 s in the TVSA 120 °C process, as shown in Figure 6d. Figure 6e compares the desorption kinetics for each condition. V-ETSA exhibited the sharpest and highest desorption rate profile, with a maximum of 0.23 mmol g^–1^ min^–1^, outperforming TVSA at 90 °C and 120 °C, which peaked at 0.09 and 0.210 mmol g^–1^ min^–1^, respectively. This enhancement is attributed to the rapid internal heat delivery enabled by Joule heating, in contrast to the slower, external heating approach used in TVSA.

When comparing the average desorption rates over the heated desorption period, V-ETSA achieved ∼2.5 times faster kinetics than TVSA at 90 °C (0.091 vs 0.036 mmol g^–1^ min^–1^). Additionally, due to poor heat transfer and greater heat loss to the PTFE housing (sensible heat) in TVSA, the energy requirement for the TVSA method is substantially higher (∼2 and 3 times higher for TVSA 120 and 90 °C, respectively) than the V-ETSA process (Figure S14). As reported by Schellevis et al., the significant contribution of the sensible heat demand from housing materials (40%) and heat transfer media (21%) during pilot-scale steam-assisted temperature vacuum swing adsorption (S-TVSA) further underscores the energetic advantage of the V-ETSA approach by reducing such parasitic heat loss.^55^

Cooling time following desorption is another critical factor influencing the overall productivity of DAC processes. As shown in Figure S15, the cooling time of the adsorbent is strongly affected by the external temperature of the module. Following desorption at 90 °C or 120 °C in the TVSA setup (Figure 6a,b), the module required approximately 3,000 s to cool down to 40 °C—about 2.7 times longer than the ∼1100 s needed in the V-ETSA process due to the higher external temperature of the module. This prolonged cooling duration further reduces the cycle frequency and, as a result, limits the overall CO_2_ capture productivity of the TVSA system. In summary, V-ETSA outperforms conventional TVSA in both CO_2_ capture productivity and energy efficiency, primarily due to its significantly shorter cycle times—enabled by faster desorption and cooling rates—as well as the direct and rapid heating of the adsorbent via Joule heating.

The V-ETSA process developed in this study achieved a desorption rate of 0.091 mmol g^–1^ min^–1^, surpassing other vacuum desorption methods reported in the relevant DAC literature (Table 1). This value exceeds those of conventional TVSA approaches, which typically range between 0.002–0.078 mmol g^–1^ min^–1^ as well as S-TVSA systems using high steam flow rates (e.g., 30.9 kg·min^–1^), which reached a maximum of 0.0625 mmol g^–1^ min^–1^.^29,55–58^ Notably, the V-ETSA system delivers these superior kinetics using Joule heating under milder temperature (85 °C) and vacuum (70 mbar) conditions without the need for external steam, highlighting it as a promising alternative for improving desorption kinetics in vacuum swing adsorption processes.

**Table 1:** Comparison of Average Desorption Rates for Different Vacuum Desorption Modes and Materials after CO_2_ Capture under DAC Conditions, as Reported in the Literature

materials	desorption method	heating method	desorption conditions	desorption rate, mmol g^–1^ min^–1^	refs
APDES-NFC-FD	TVSA	heating jacket	50 mbar, 95 °C	0.008	^56^
amine/silica gel	TVSA	heating bath	50 mbar, 90 °C	0.002	^29^
proprietary aminoresin	TVSA	heating/cooling baths	11 mbar, 60 °C	0.019	^57^
PEI/MCF	TVSA	heating tape	120 mbar, 100 °C	0.004	^58^
PEI/MCF	S-TVSA	30.9 kg_s_^–1^ min^–1^ steam	120 mbar, 100 °C	0.0625	^58^
Lewatit VP OC 1065	S-TVSA	0.86 g kg_s_^–1^ min^–1^ steam	74 mbar, 110 °C	0.023	^55^
PEI/GO foam	TVSA	heating tape	70 mbar, 120 °C	0.078	this study
PEI/GO foam	TVSA	heating tape	70 mbar, 90 °C	0.036	this study
PEI/GO foam	V-ETSA	Joule heating	70 mbar, 85 °C	0.091	this study

Figure 6f presents the cyclic CO_2_ desorption capacities and purities of PEI2.5­(25k)-TAGO900 across successive V-ETSA cycles, which we used to assess the cyclic stability of the adsorbent. A gradual decline in capacity was observed up to the 25th cycle, corresponding to a degradation rate of approximately 0.02 mmol g^–1^ cycle^–1^. This loss in performance is hypothesized to result from several contributing factors: (i) partial evaporation and removal of PEI25k due to its non-negligible vapor pressure (PEI25k exists as a viscous liquid at 25 °C), (ii) oxidative degradation of PEI due to a minor air leakage (6.8 mbar min^–1^ air, corresponding ∼200 ppm min^–1^ O_2_) during the vacuum desorption step, or (iii) a combination of these mechanisms. Morphological degradation, such as aggregation of hydrophilic PEI on the hydrophobic surface of the rGO framework, was excluded as a potential cause of the decreasing CO_2_ desorption capacity due to the intact morphology of PEI25k before and after 10 V-ETSA cycles, as shown via electron microscopy in Figure S16. In the literature, similar degradation phenomena during TVSA processes have been reported, where capacity loss is attributed to the volatilization or oxidation of amine-functionalized materials.^57–60^ For instance, Leenders et al. suggested that the CO_2_ adsorption capacity of Lewatit VP OC 1065, a benzyl amine-based adsorbent, can be degraded (80% of its original capacity) when exposed to a 100 ppm level of O_2_ at 120 °C for ∼ 800 h.^60^ The accelerated degradation rate observed in less viscous PEI5(800)-TAGO900 (∼0.04 mmol g^–1^ cycle^**–1**^, Figure S17) further supports the hypothesis that PEI loss is governed by vapor pressure and/or oxidative degradation since PEI800, with higher volatility and lower viscosity than PEI25k, is more susceptible to evaporation and oxidative degradation (faster O_2_ diffusion) under V-ETSA conditions. Although oxidatively degraded volatile amine species were not detected, X-ray photoelectron spectroscopy (XPS) survey analysis indicated a decrease in the surface amine content and an increase in the surface oxygen content after 10 V-ETSA cycles, with more pronounced loss observed in PEI5(800)-TAGO900 than in PEI25k-based samples, as shown in Figure S18. For PEI2.5­(25k)-TAGO900, we hypothesize that the adsorbent undergoes thermal stabilization and reaches a morphologically stable state after approximately 25 cycles. While these observations provide an insight into the degradation mechanisms occurring during V-ETSA, a comprehensive mechanistic investigation—including tracking the stepwise chemical transformation of the amine species—is beyond the scope of this study. Further research is needed to fully elucidate these degradation pathways and to inform the design of more robust amine-based adsorbents for long-term operation under V-ETSA conditions. In future work, strategies such as cross-linking the amines,^61,62^ functionalizing amines with epoxides,^63^ and/or minimizing operational vacuum leaks could be explored to suppress amine evaporation and improve the thermal and oxidative stability of the sorbents during cyclic V-ETSA. Notably, after 25 cycles, PEI2.5­(25k)-TAGO900 exhibited stable performance, with desorption capacity losses falling below 0.01 mmol g^–1^ cycle^–1^ and stabilizing around 0.94 mmol g^–1^.

In addition to desorption capacities and kinetics, CO_2_ purity is an important yet often underreported metric in DAC system evaluations. Based on a Web of Science database search with the keywords “direct air capture” and “vacuum”, among the more than 100 relevant DAC studies using vacuum desorption, only a few papers explicitly reported the purity of the desorbed CO_2_ stream, with reported values ranging from 50% to 99%.^29,55,64–67^ For example, Wurzbacher et al. demonstrated a CO_2_ purity as high as 99%, whereas Schellevis et al. reported lower purity (50–55%), due to vacuum leaks during desorption.^29,55^ Accurate determination of CO_2_ purity can be influenced by various factors, including vacuum leaks, system scale, and the relative ratio of dead volume to adsorbent volume. Nonetheless, within the same experimental configuration, meaningful comparisons of desorption performance are possible. Figure 6f presents the CO_2_ purity observed during cyclic V-ETSA operations. Over 36 cycles, the desorbed CO_2_ purity (defined as the ratio of desorbed CO_2_ to the sum of desorbed CO_2_ and N_2_) decreased gradually from 83% to 77%, primarily due to the decreased CO_2_ desorption capacity over the cycles. In Figure S23, compared with conventional TVSA at 90 °C, V-ETSA delivered higher CO_2_ purity (70% vs 77%, respectively), owing to its greater desorption capacity and faster desorption kinetics, which reduced the extent of dilution from leaked N_2_ from outside atmosphere.

## 3 Conclusions

The electrically-conductive amine-functionalized rGO foam presented here exemplifies a multifunctional material tailored for electrically-driven thermal CO_2_ desorption in DAC. This structured architecture serves not only as an adsorbent but also as a built-in heater, potentially paving the way for energy efficient and responsive DAC modules for vacuum-assisted electrical thermal swing adsorption (V-ETSA) systems. The combination of hydrothermal synthesis and thermal annealing facilitated fast and direct heating of the adsorbent foam without using additional chemicals. After amine infusion with water as a solvent, the V-ETSA process demonstrated superior desorption kinetics, desorbed CO_2_ purity, and energy efficiency compared to the conventional TVSA processes. Although the PEI2.5­(25k)-TAGO900 adsorbent exhibited a gradual decrease in CO_2_ desorption capacity at a rate of 0.02 mmol g^–1^ cycle^–1^ over the first 25 cycles, it retained a capacity of 0.94 mmol g^–1^ after 25 cycles. In contrast, the PEI800-functionalized TAGO900 showed a faster degradation rate and greater nitrogen loss, indicating higher susceptibility to amine evaporation and oxidation. These observations underscore the influence of polymer volatility and viscosity under V-ETSA conditions (0.07 bar vacuum, ∼85 °C, and a vacuum leak rate of 6.8 mbar min^–1^). A detailed mechanistic investigation is beyond the scope of this work, but future studies should explore approaches such as polymer cross-linking, covalent anchoring, or tailored surface chemistry to enhance capacity retention under rapid cycling.

Overall, this study presents a pathway for accelerating CO_2_ desorption rates in DAC and related adsorption-based separation processes. Future work building on this initial study should include parametric experiments for better V-ETSA process optimization,^38^ incorporation of the electrically conductive materials into mechanically stronger structures using polymeric binders,^39,68^ and industrial-scale long-term stability experiments (∼1000 cycles).

## 4 Experimental Section

### 4.1 Materials

Powder-type reduced graphite oxide (rGO) and solution of graphite oxide (GO) dispersed in water (1.0 wt %) were purchased from Angstron Materials. Poly­(ethylenimine) (PEI) with molecular weights of 800 and 25k were provided by Sigma-Aldrich. Ultrahigh purity gases (99.999% N_2_, 99.999% Ar, and 400 ppm of CO_2_/N_2_) were obtained from Airgas. Chemicals and gases were used without further purification.

### 4.2 Sample Preparation

#### 4.2.1 GO Foam Synthesis

Graphene oxide (GO) hydrogel synthesis was adapted from Xu et al.^41^ Aqueous GO solution (5 or 10 mg/mL) was dispersed using horn-type sonicator for 30 min. The GO aqueous dispersion (20 mL) was added into a Teflon liner (47 mL), and the hydrothermal reactor was assembled. The assembled hydrothermal reactor was placed in a convection oven at 200 °C. After 15 h, the hydrothermal reactor naturally cooled down to room temperature. The resulting GO hydrogel was further lyophilized at −50 °C for 24 h to obtain hydrothermally fabricated GO foam (HTGO).

#### 4.2.2 Thermal Annealing of HTGO Foam

As-synthesized HTGO was further annealed at elevated temperatures to obtain higher electrical conductivity. The HTGO was placed in a tube furnace, and the furnace was filled with Ar (200 sccm). After oxygen level below 0.5 ppm by oxygen sensor (Rapidox 1100, Cambridge Sensotec), the furnace temperature started to increase by 5 °C min^–1^ up to 110 °C and degassed for 4 h. Subsequently, the temperature ramped up to desirable temperatures (500–900 °C) at a rate of 2 °C min^–1^ and was held at the final temperature for 8 h for annealing, followed by natural cooling to acquire thermally annealed GO (TAGO).

#### 4.2.3 Amine Functionalized TAGO Foam Synthesis

A structured TAGO was functionalized by the amine infusion method as similar to our previous publication.^64^ First, a vial (20 mL) fully charged with a certain amine concentration (2.5–20 wt %) in D.I. water was prepared. Bath-type sonication was allowed for 20 min to completely dissolve amines. The fabricated GO foam was immersed in the vial with amine solution, and the vial was sealed with parafilm to prevent solution leakage. The vial with GO foam was placed in a roller for 4 h to achieve homogeneous amine functionalization. Finally, the amine functionalized TAGO was freeze-dried at −50 °C for 24 h to remove residual water.

#### 4.2.4 Material Characterizations

Microscopic photographs of the studied samples were captured using scanning electron microscopy (SEM) (SU8230, Hitachi). N_2_ (77 K) and CO_2_ (273.15 K) physisorption experiments (Autosorb IQ, Anton Paar) were performed to reveal meso- and micro porosities of the synthesized samples. Before the physisorption measurement, each sample (70 mg) was activated at 80 °C for 2 h under 0.1 mbar vacuum. Powder X-ray diffraction (PXRD) patterns were analyzed using Rigaku Miniflex (Cu Kα radiation) to calculate the *d*-spacing of the samples. Raman spectra (QONTOR Confocal Raman, Renishaw) were collected with a 488 nm laser to compare I_d_ and I_g_ ratios of the synthesized samples. Bulk chemical compositions (C, H, and N) were determined via elemental analysis (EA) at Atlantic Microlab (Norcross, GA, USA). The oxygen content is estimated by residual weight fraction after EA X-ray photoelectron spectroscopy (XPS) (Thermo Kα, Thermo Fisher Scientific) was used to assess the surface chemical compositions of the samples. The modulated dynamic scanning calorimetry (MDSC) method and the sapphire method were used to obtain the heat capacities of the synthesized materials using a dynamic scanning calorimeter (DSC) (DSC250, TA Instrument).

#### 4.2.5 Electrical Resistance and Joule Heating Measurement

The electrical resistance of the synthesized GO foams was measured by a Keithley 2400 SMU (Tektronix). The dimensions of GO structures were 1.0 cm in diameter and 1.5 cm in length. During the Joule heating experiment, voltage was supplied, and the corresponding current was measured by (R-SPS3010, Nice-Power). The temperature of the adsorbent was monitored and recorded by Platinum software and CS8DPT PID controller (Omega Engineering).

### 4.3 Gas Adsorption

#### 4.3.1 Gravimetric Gas Adsorption

Gravimetric gas adsorption experiments under dry and humid 400 ppm of CO_2_/N_2_ were conducted using thermogravimetric analysis at 30 °C (TA Instruments, Q-500 and Q-5000). Several pieces of amine functionalized GO foam, amounting to ∼5 mg, were placed in a platinum pan. The sample was activated at 110 °C for 2 h under 90 sccm dry N_2_ before adsorption started. For dry experiments, dry 400 ppm of CO_2_ was flowed at 90 sccm under 30 °C. In case of humid experiments, the sample was presaturated for 6 h by 100 sccm 50% RH (30 °C) N_2_ flow before CO_2_ adsorption. Then, humid 400 ppm of CO_2_/N_2_ gas stream was introduced after presaturation, and the weight change was monitored to calculate the adsorbed CO_2_ amount.

#### 4.3.2 Water Isotherms

Water isotherms for the synthesized materials were measured using a volumetric equilibrium adsorption instrument (Vstar, Anton Paar). About 70 mg of the samples were degassed at 80 °C for 2 h under 0.1 mbar vacuum before the adsorption measurement. Two equilibrium conditions were used to facilitate adsorption experiments. In the relative pressure range ≤0.012 P/P_0_, equilibrium was evaluated every 60 s with pressure threshold 0.05 Torr/min. Above 0.012 P/P_0_, the pressure threshold was changed to 0.01 Torr/min with the same evaluation interval. For each dose, maximum equilibration time was set to 60 min.

#### 4.3.3 Fixed Bed Adsorption and Temperature-Programmed Desorption (TPD)

Amine functionalized TAGO was loaded in a stainless-steel bed (3/8-in. tube diameter; 0.035-in. wall thickness). Both ends were packed with glass wool, and the bed was connected to a custom-built fixed bed as depicted in our previous report.^64^ The temperature of the system was controlled by a chiller (Julabo, 600F) for adsorption and desorption. The degassing of the sample was conducted at 80 °C for 2 h with dry N_2_. For dry CO_2_ adsorption, dry 400 ppm of CO_2_/N_2_ was flowed to the adsorbent bed under a desirable adsorption temperature ranging from −15–40 °C. For humid CO_2_ adsorption, wet 400 ppm of CO_2_/N_2_ with the desired humidity (20–70% RH) was introduced without presaturation of the sample with water. Breakthrough (BT) and pseudo equilibrium (PE) capacities were defined as the amount of adsorbed CO_2_ until the ratio of outlet and inlet CO_2_ concentration (C/C_0_) reads 5 or 95%, respectively. After adsorption, temperature-programmed desorption (TPD) was performed by increasing the temperature at 0.5 °C/min up to 80 °C and monitoring desorbed CO_2_ and water concentrations. The outlet gas concentration was measured by an IR analyzer (LI-COR, LI-850).

#### 4.3.4 Vacuum-Assisted Electrical Thermal Swing Adsorption (V-ETSA) and Temperature Vacuum Swing Adsorption (TVSA)

Amine-functionalized, conductive GO foams (TAGO) were assembled into a V-ETSA module using an electrically conductive adhesive (AA-Duct 906, Atom Adhesives). Nonconductive materials, such as PTFE (1/2-in. tube diameter), were used for the module’s housing. The V-ETSA and TVSA processes each consist of four stages: a vacuum purge (5 min to remove interstitial gas at the adsorption temperature), desorption (85–120 °C), cooling (to 40 °C), and adsorption (22 °C and 70% RH). The initial degassing step was performed under an N_2_ atmosphere with Joule heating to fully activate the adsorbent materials. Beginning with the second desorption cycle, a 0.07 bar vacuum (Hiscroll 6, Pfeiffer) was used to desorb the adsorbed species, which were monitored by a mass spectrometer (GSD350, Pfeiffer). Although TPD experiments under N_2_ indicated that water desorbed at a lower temperature (desorption peak at 25 °C) than CO_2_ (desorption peak at 64 °C), water was not detected by the mass spectrometer—likely due to condensation in cold spots within the vacuum manifold, such as the vacuum pump. The module’s internal and external temperatures during desorption were recorded using Platinum Monitor software (CS8DPT, Omega Engineering) and thermocouples connected to a PID controller (CS8DPT, Omega Engineering). Electrical power was supplied by a source meter (R-SPS3010, Nice-Power) to provide Joule heating. Typically, a voltage range of 4–5 V was used to regulate the internal temperature between 75–90 °C. In the case of TVSA, a heating tape was used to raise the module temperature to the desired values (90 °C or 120 °C). Adsorption was carried out using simulated air (400 ppm CO_2_ in N_2_) at 70% relative humidity (22 °C), controlled by a dew point generator (LI-610, LI-COR).

## Supplementary Material



## Data Availability

Data supporting this article are provided in the Supporting Information (SI). Raw data can be obtained from the corresponding author upon reasonable request.

## Notes

Summary of EA analysis results; Parameters for thermal energy requirement calculations; Pore size distribution of the studied samples from N_2_ physisorption, and Desorbed gas concentration profiles (PDF)
