# Supplementary material for: Electrically Conductive Amine Functionalized Reduced Graphite Oxide Foam for CO2 Removal from the Air
Source: ACS Appl Mater Interfaces. 2025 Nov 18;17(48):65565–76. doi: 10.1021/acsami.5c15229 (PMC12679536; doi:10.1021/acsami.5c15229)
Supplement: Supplementary file 1 [file am5c15229_si_001.pdf]

Supporting Information

Electrically Conductive Amine Functionalized Reduced Graphite Oxide Foam for CO_2 Removal from the Air

MinGyu Song, Jaedeok Kim, Christopher W. Jones*, Ryan P. Lively*

School of Chemical & Biomolecular Engineering, Georgia Institute of Technology, Atlanta, GA 30332

Emails: cjones@chbe.gatech.edu, ryan.lively@chbe.gatech.edu

**Contents**	**Description**	**Page #**
**Table S1**	Summary of EA analysis results	S3
**Table S2**	Textural properties of selected rGO foams	S3
**Table S3**	Parameters for thermal energy requirement calculations	S3
**Figure S1**	CO_2 uptake of PEI impregnated GO powder using TGA	S4
**Figure S2**	Photographs of HTGO hydrogels	S5
**Figure S3**	Photographs of various rGO	S6
**Figure S4**	XRD and Raman spectra of rGO foams	S7
**Figure S5**	CO_2 uptake of PEI5(800)-TAGO900 from breakthrough experiments	S8
**Figure S6**	Cross-section SEM photographs of rGO foams	S9
**Figure S7**	N_2 physisorption (77 K) results of the studied samples	S10
**Figure S8**	Pore size distribution of the studied samples from N_2 physisorption	S11
**Figure S9**	CO_2 isotherms (273 K) and pore size distribution of HTGO and TAGO900	S12
**Figure S10**	CO_2 (400 ppm balanced with N_2) breakthrough profiles of PEI5(800)-TAGO900	S13
**Figure S11**	CO_2 (400 ppm balanced with N_2) breakthrough profiles of PEI2.5(25k)-TAGO900	S14
**Figure S12**	CO_2 (400 ppm balanced with N_2) breakthrough profiles of PEI5(25k)-TAGO900	S15
**Figure S13**	V-ETSA module and diagram for custom V-ETSA apparatus	S16
**Figure S14**	Relative thermal energy requirements of the studied desorption processes	S17
**Figure S15**	Comparison of cooling time after V-ETSA desorption of PEI2.5(25k)-TAGO900 at different module temperatures	S18
**Figure S16**	SEM images of PEI2.5(25k)-TAGO900 (a) before and (b) after 10 V-ETSA cycles	S19
**Figure S17**	CO_2 desorption capacities and purities for PEI5(800)-TAGO900 and PEI5(25k)-TAGO900 samples during V-ETSA cycles	S20
**Figure S18**	Nitrogen and oxygen content of PEI5(800)-TAGO900 & PEI2.5(25k)-TAGO900 by XPS survey before/after V-ETSA cycles	S21
**Figure S19**	XPS survey spectrum for PEI5(800)-TAGO900 and PEI2.5(25k)-TAGO900 before and after 10 V-ETSA cycles	S22
**Figure S20**	Water isotherms of TAGO900 and PEI2.5(25k)-TAGO900 at 25 °C	S23
**Figure S21**	Pressure drops of PEI2.5(25k)-TAGO900 foams	S24
**Figure S22**	Specific heat capacity of the standard sapphire sample and PEI2.5(25k)-TAGO900	S25
**Figure S23**	Desorbed gas concentration profiles	S26
	**References**	S27

Table S1. Summary of EA analysis results for the rGO foams discussed in this study

	C, wt%	N, wt%	H, wt%	N/C ratio
HTGO	77	0.3	0.8	-
TAGO500	85	0.1	0.9	-
TAGO700	90	0.1	0.9	-
TAGO800	93	0.1	0.9	-
TAGO900	95	0.1	1.0	-
PEI5(800)-TAGO900	71	11.3	4.7	0.15
PEI10(800)-TAGO900	67	17.1	6.7	0.26
PEI20(800)-TAGO900	59	22.1	8.4	0.37
PEI2.5(25k)-TAGO900	71	13.1	5.6	0.19
PEI5(25k)-TAGO900	67	15.8	6.5	0.24
PEI10(25k)-TAGO900	65	19.7	8.6	0.30

Table S2. Textural properties of selected rGO foams discussed in this study

	d-spacing, Å	Crystallite size, nm	BET Surface area, m^2/g	Pore volume, mL/g
HTGO	3.55	16.7	188	0.29
TAGO500	3.43	16.3	346	0.36
TAGO700	3.40	15.7	428	0.43
TAGO900	3.37	13.5	410	0.45
PEI2.5(25k)-TAGO900	-	-	104	0.12
PEI5(25k)-TAGO900	-	-	43	0.05

* Bragg equation (XRD) and Tuinstra Koenig relation[1] (Raman spectroscopy) were used to calculate d-spacing and crystallite size of the rGO samples.

Table S3. Parameters for thermal energy requirement calculations

Parameters	TVSA 90 °C	TVSA 120 °C	V-ETSA 85 °C
Swing temperature for the adsorbent, °C	43	62	63
Swing temperature for the housing, °C	68	98	25
Mass of the adsorbent, g	0.12	0.12	0.12
Mass of the housing material, g	14.38	14.38	14.38
Working CO_2 capacity, mmol/g	0.6	1.3	0.94
Working H_2O capacity[a], mmol/g	18.89	18.89	18.89
Specific heat capacity of CO_2[2], J/g/°C	0.844	0.844	0.844
Specific heat capacity of H_2O[2], J/g/°C	4.184	4.184	4.184
Specific heat capacity of the adsorbent[b], J/g/°C	0.687	0.687	0.687
Specific heat capacity of the housing[3], J/g/°C	1.023	1.023	1.023
Latent heat of CO_2, kJ/mol[4]	104	104	104
Latent heat of H_2O, kJ/mol[4]	46.4	46.4	46.4

a: The H_2O working capacity at 22 °C and 70% RH was estimated from the adsorption isotherm value (**Fig. S20**) and complete desorption. The working capacity of H_2O during V-ETSA operation could not be accurately quantified due to condensation occurring at multiple cold spots along the vacuum manifold, including within the vacuum pump.

b: The specific heat capacity of the adsorbent was measured in **Fig. S22**.

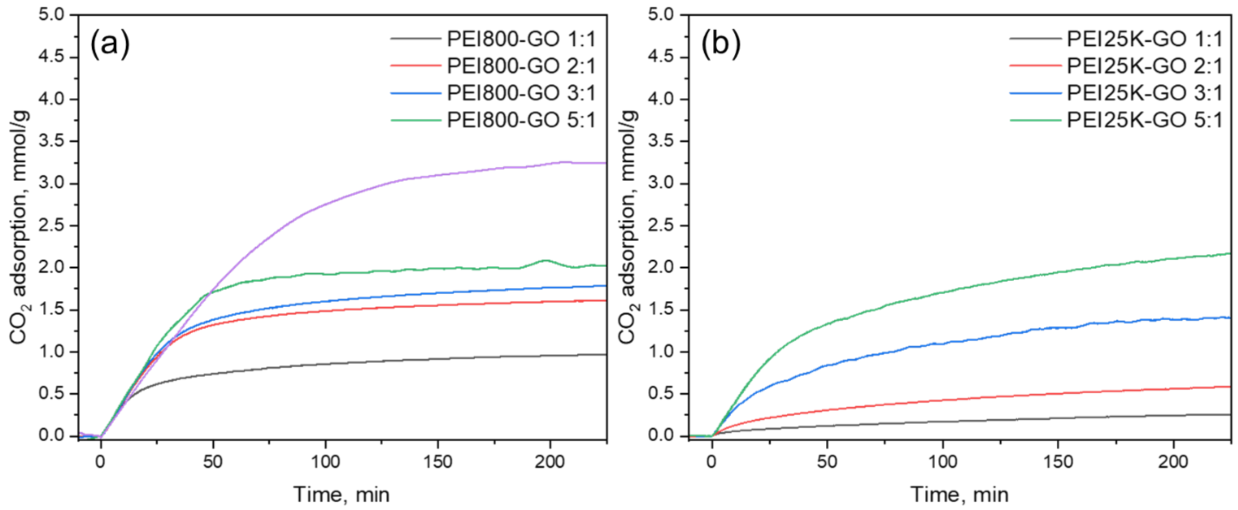

Figure S1. CO_2 uptake of branched poly(ethyleneimine) (PEI) impregnated graphite oxide powder under 400 ppm CO_2/N_2 and 50% RH at 30 °C using the gravimetric method. Molecular weights of PEI were (a) 800 and (b) 25k Da. Weight ratios of amine and graphene oxide are presented in the sample name. Regardless of the molecular weight, more amines result in higher CO_2 uptake under the tested adsorption conditions.

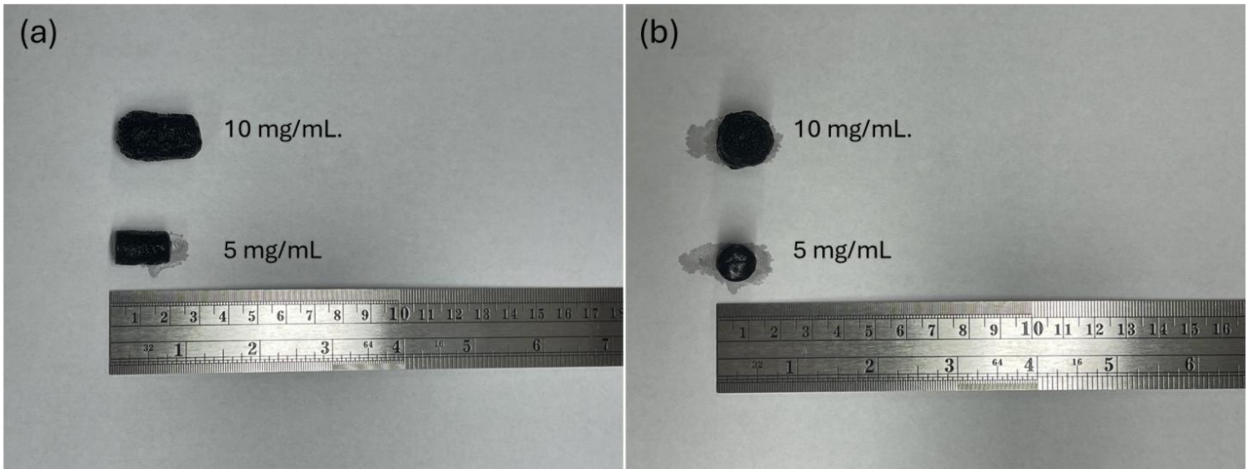

Figure S2. (a) Side and (b) top view of hydrothermally treated reduced graphite oxide (HTGO) hydrogel with different graphite oxide (GO) concentration during hydrothermal reaction. The size of the HTGO can be controlled by the size of the reactor and the GO concentration.

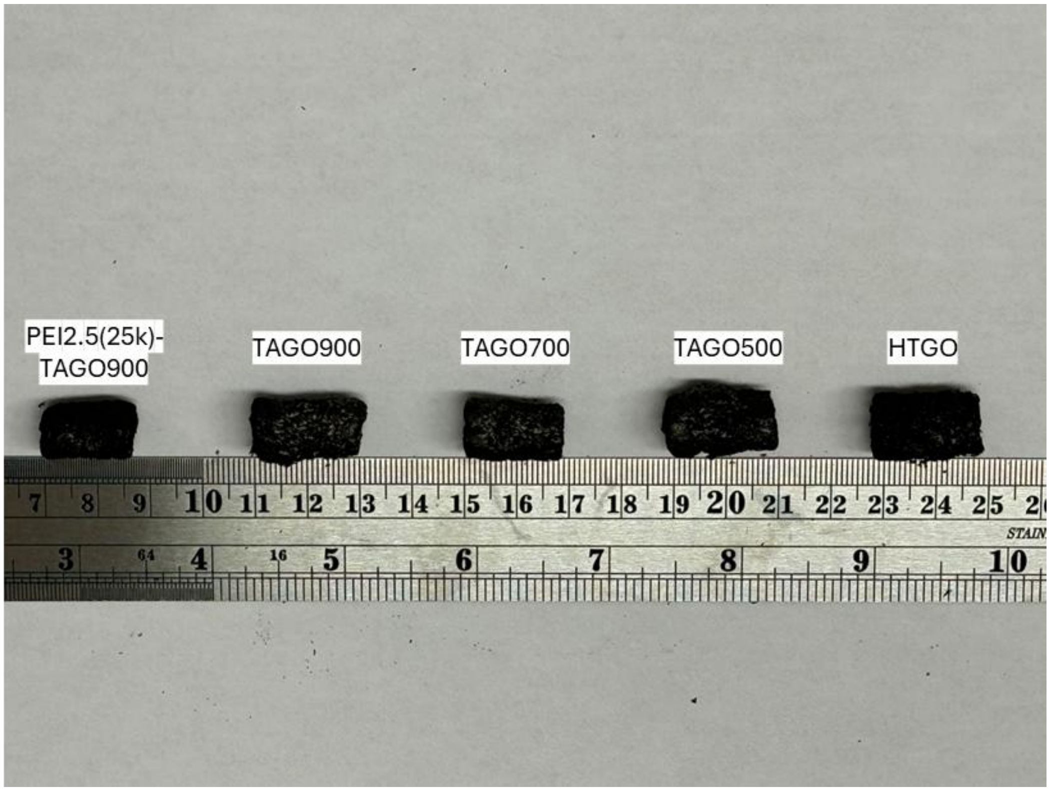

Figure S3. Photographs of different reduced GO (rGO) foams. From left, PEI (40 wt%) infused rGO foam after thermal treatment at 900 °C (PEI2.5(25k)-TAGO900), thermally treated RGO foams at 700 and 500 °C (TAGO700 and TAGO500), and hydrothermally synthesized GO foam before thermal treatment (HTGO). The shape and size of the foams are intact before and after amine infusion and thermal treatments.

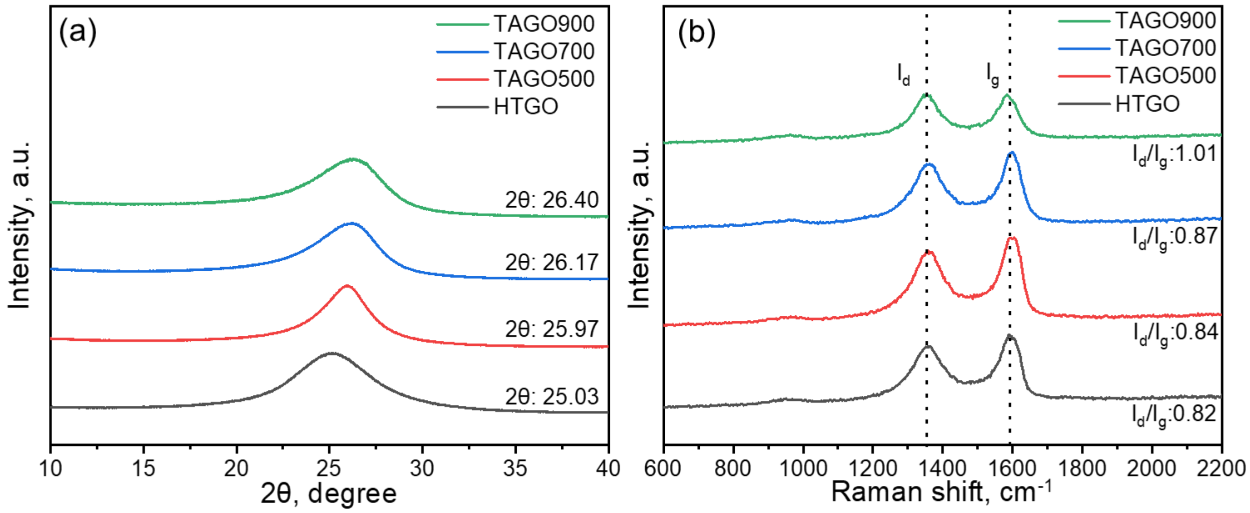

Figure S4. (a) XRD and (b) Raman spectra of rGO foams investigated in this study. D-spacing and crystal sizes estimated by the Bragg equation and the Tuinstra Koenig relation[1] are available in **Table S2**.

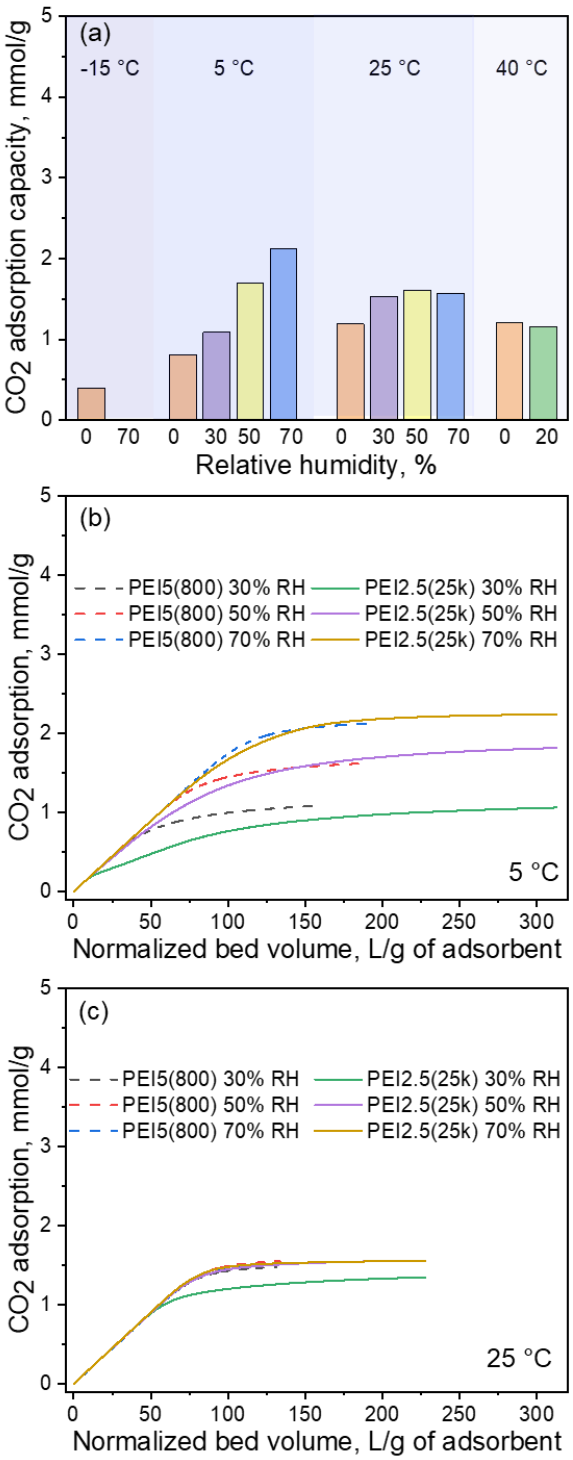

Figure S5. (a) Summary CO_2 uptake of PEI5(800)-TAGO900 from 400 ppm CO_2 (N_2 balance) breakthrough experiments and (b and c) CO_2 adsorption profiles at 5 and 25 °C. Adsorbents with higher amine molecular weight results in slower CO_2 uptake rate at 5 °C, likely due to higher diffusion barrier in the viscous PEI 25k Da film. However, the difference in adsorption kinetics between PEI25k and PEI800 decreased at higher humidity and temperature conditions, due to improved amine mobility and reduced diffusion barriers.

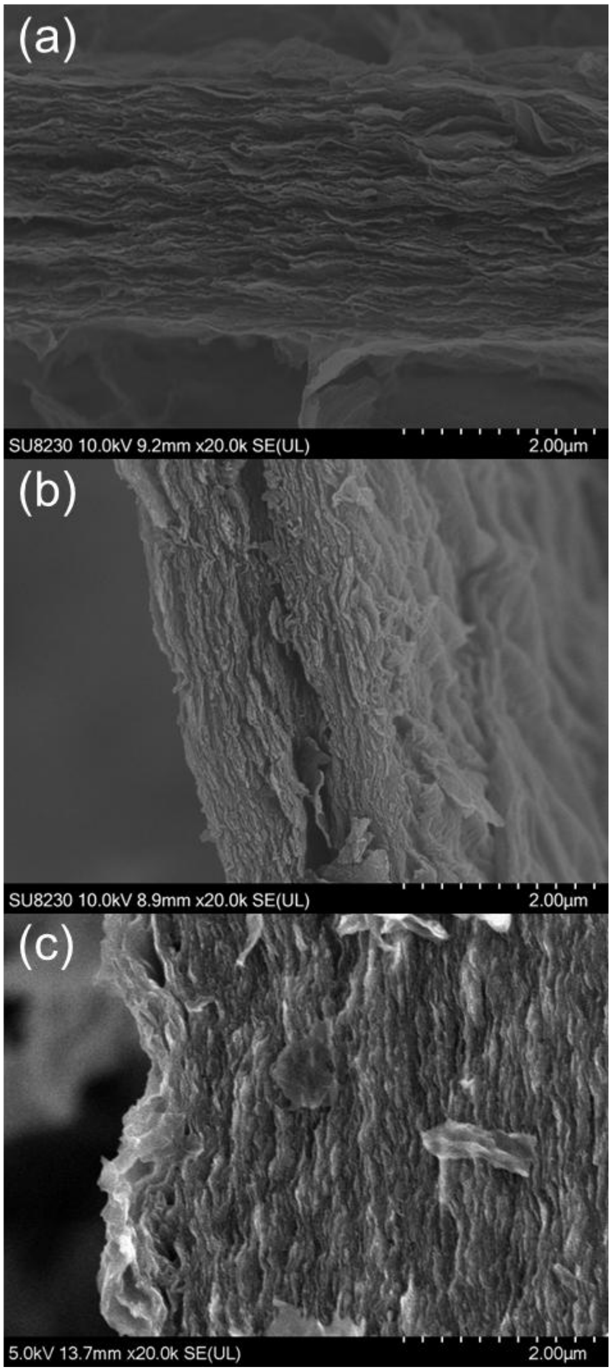

Figure S6. Cross-sectional SEM images of (a) HTGO, (b) TAGO900, (c) PEI2.5(25k)-TAGO900. The layer-by-layer structure of rGO is maintained after thermal treatment and amine infusion.

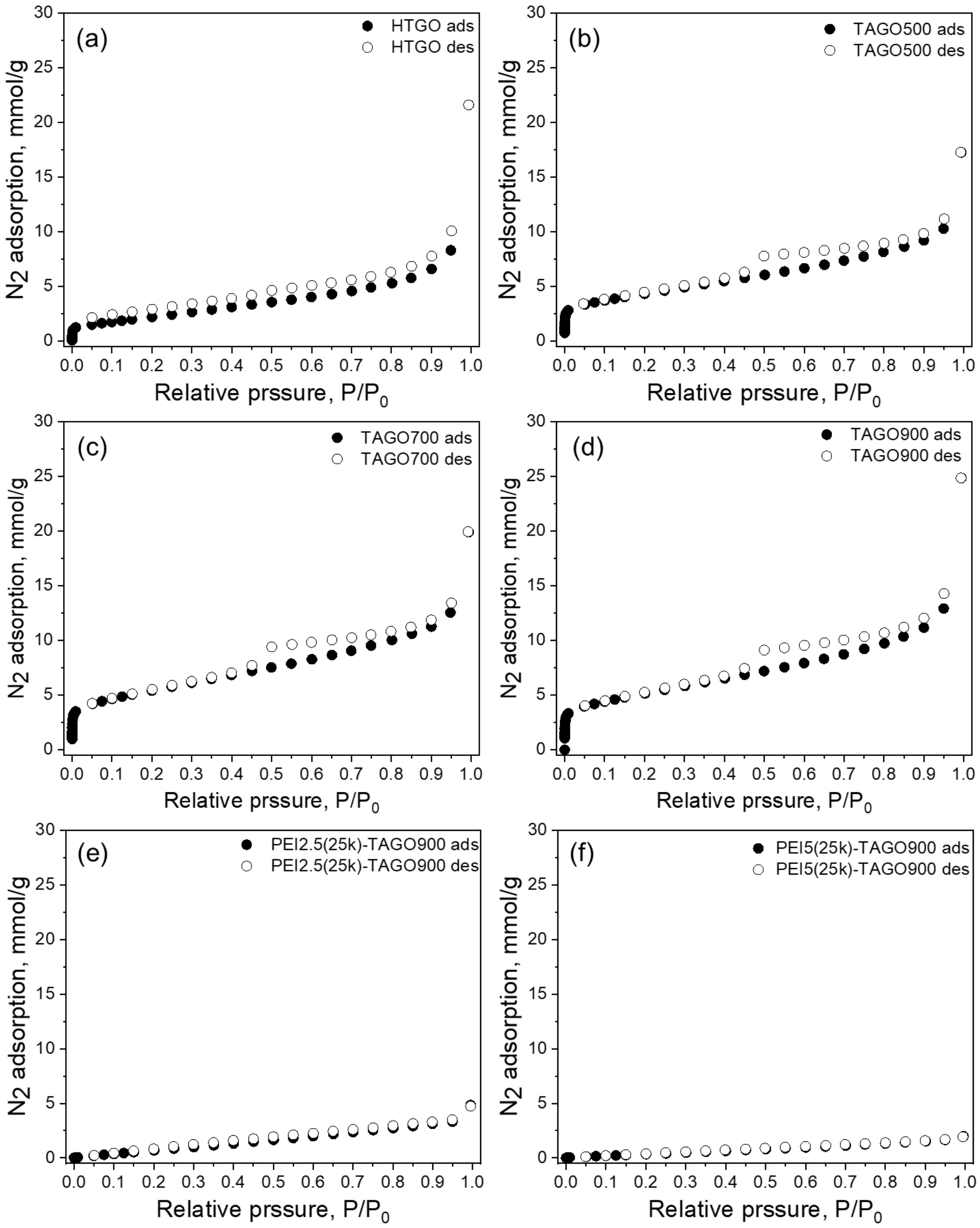

Figure S7. N_2 physisorption (77 K) results of the (a) HTGO, (b) TAGO500, (c) TAGO700, (d) TAGO900, (e) PEI2.5(25k)-TAGO900, and (f) PEI5(25k)-TAGO900. Thermal annealing improved N_2 physisorption of HTGO, but amine impregnation blocked the majority of the N_2 accessible pores.

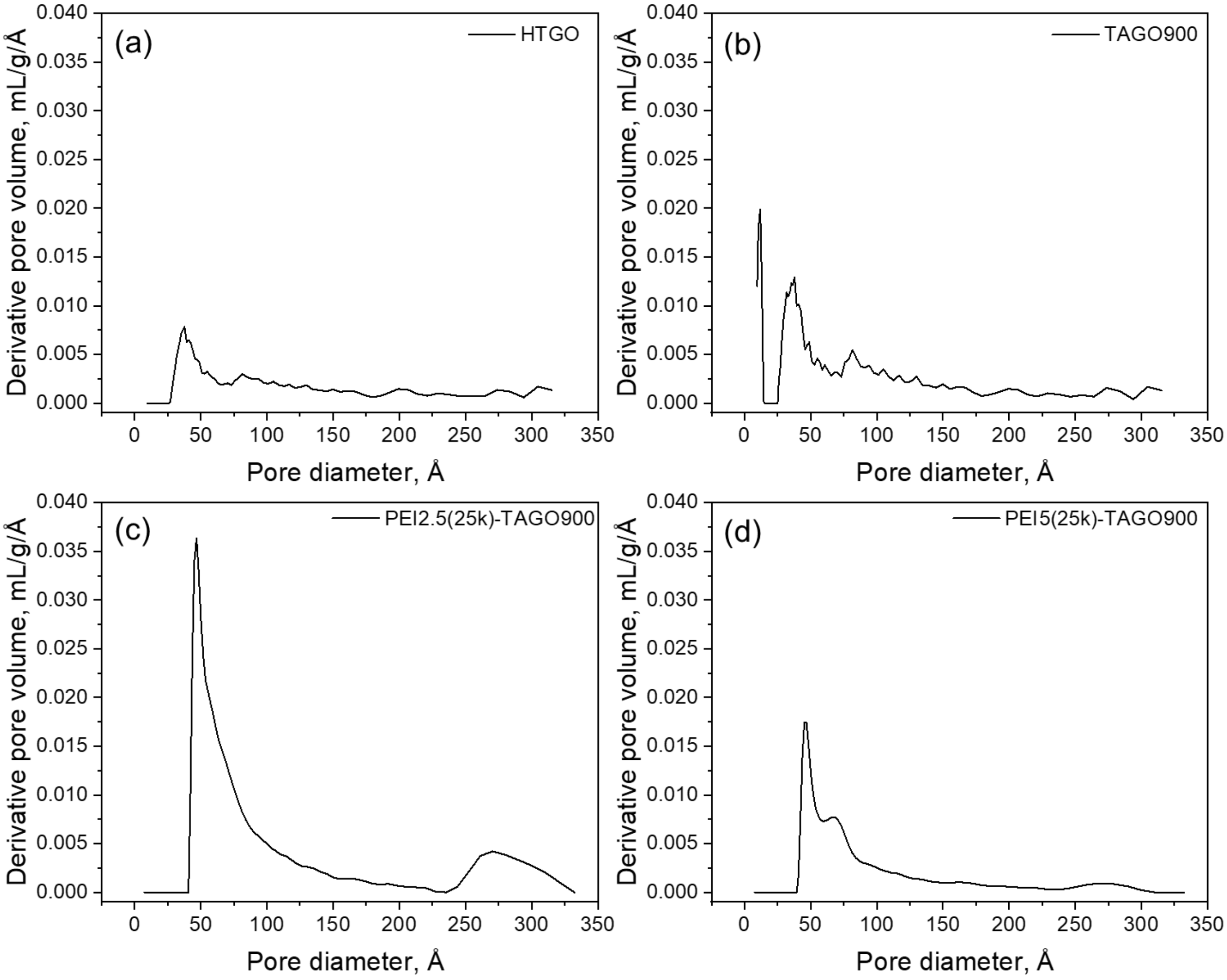

Figure S8. Pore size distribution of (a) HTGO, (b) TAGO900, (c) PEI2.5(25k)-TAGO900, and (d) PEI5(25k)-TAGO900 from N_2 physisorption. Quenched solid density functional theory (QSDFT) method for carbonaceous slit and sphere pores built-in Quantachrome software was used. Thermal annealing improved both the micro- and mesoporosity of the HTGO. PEI infusion increased the mesopore volume of PEI2.5(25k)-TAGO900, likely due to the pores created during lyophilization. However, the decreased mesopore volume with further loading of PEI25k suggests that a higher concentration of PEI25 interrupts the formation of small ice crystals.

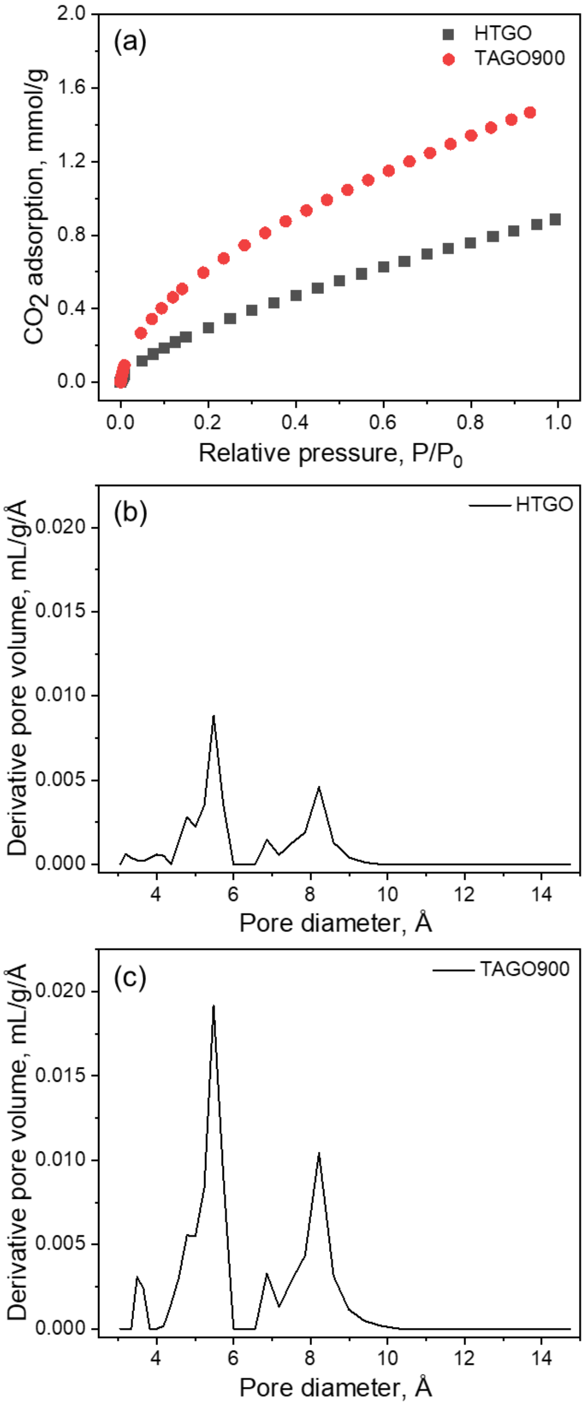

Figure S9. (a) CO_2 isotherm (273 K) results before and after thermal annealing of HTGO and (b and c) their pore size distribution. Thermal annealing improved the microporosity of the HTGO foam. Non-local density functional theory (NLDFT) method built-in Quantachrome software for carbon material was used to analyze the pore size distribution.

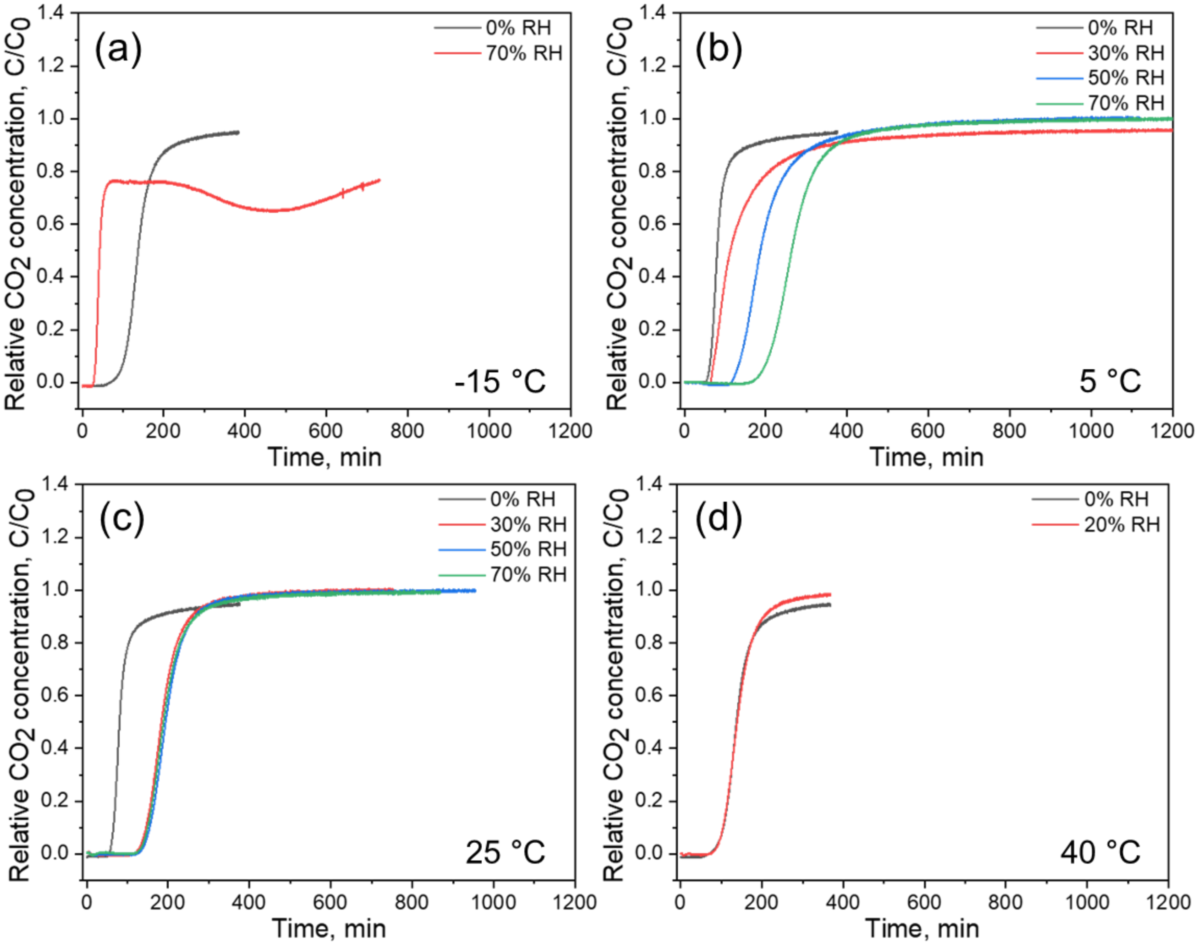

Figure S10. CO_2 (400 ppm balanced with N_2) breakthrough profiles of PEI5(800)-TAGO900 at (a) -15, (b) 5, (c) 25, and (d) 40 °C. The flow rate was 90 sccm during adsorption. Desorption was conducted at 80 °C for 2 h using 130 sccm of 99.9% N_2. A distinct dip in the CO_2 breakthrough profile at -15 °C and 70% RH was observed concurrently with water breakthrough. This feature likely arises from reduced competitive adsorption between CO_2 and H_2O under these conditions and/or an alleviation of diffusional constraints following hydration and subsequent swelling of the amine-containing layer.

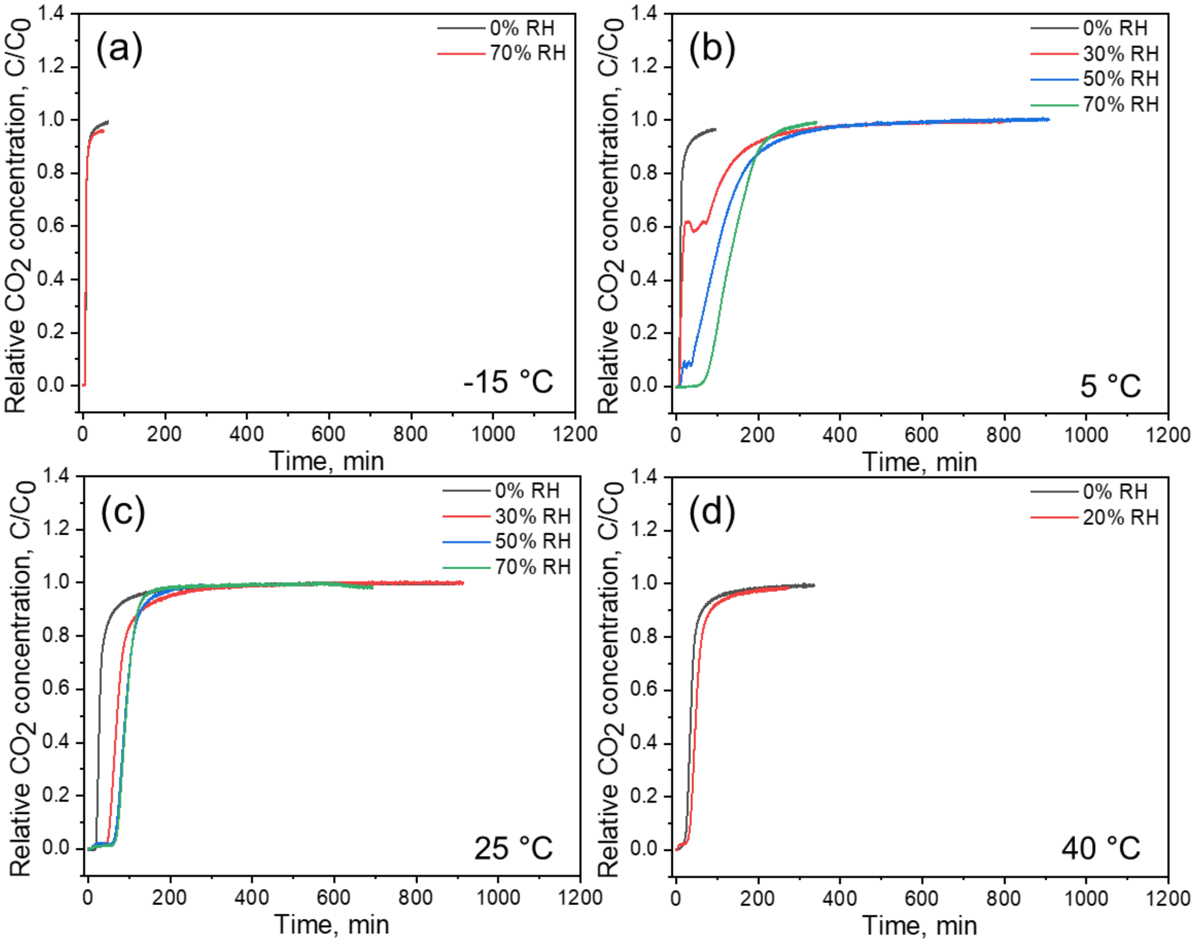

Figure S11. CO_2 (400 ppm balanced with N_2) breakthrough profiles of PEI2.5(25k)-TAGO900 at (a) -15, (b) 5, (c) 25, and (d) 40 °C. The flow rate was 40 sccm during adsorption. Desorption was conducted at 80 °C for 2h using 60 sccm of 99.9% N_2. A distinct dip in the CO_2 breakthrough profile at 5 °C under 30 and 50% RH was observed concurrently with water breakthrough. This feature likely arises from reduced competitive adsorption between CO_2 and H_2O under these conditions and/or an alleviation of diffusional constraints following hydration and subsequent swelling of the amine-containing layer.

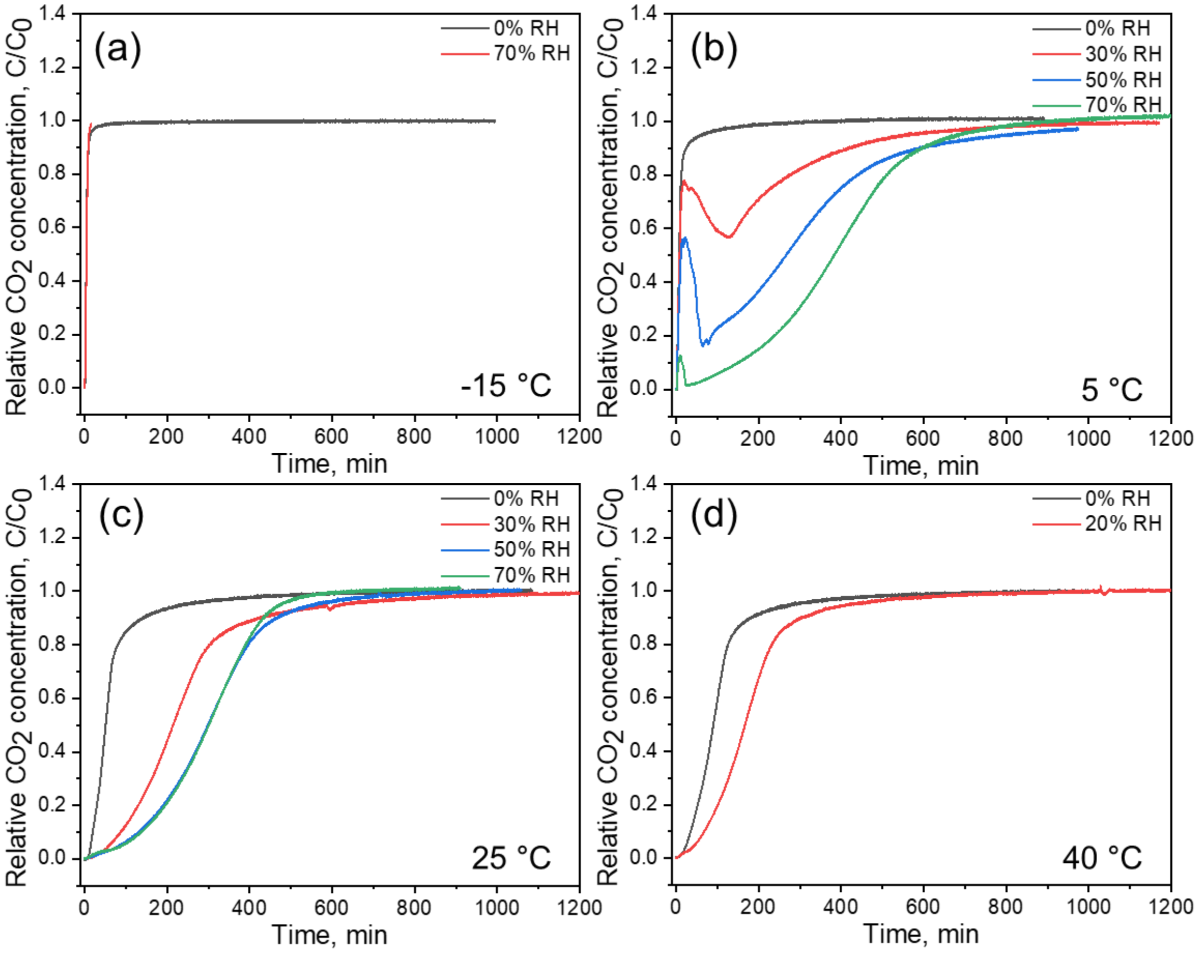

Figure S12. CO_2 (400 ppm balanced with N_2) breakthrough profiles of PEI5(25k)-TAGO900 at (a) -15, (b) 5, (c) 25, and (d) 40 °C. The flow rate was 40 sccm during adsorption. Desorption was conducted at 80 °C for 2h using 60 sccm of 99.9% N_2. A distinct dip in the CO_2 breakthrough profile at 5 °C under humid conditions was observed concurrently with water breakthrough. This feature likely arises from reduced competitive adsorption between CO_2 and H_2O under these conditions and/or an alleviation of diffusional constraints following hydration and subsequent swelling of the amine-containing layer.

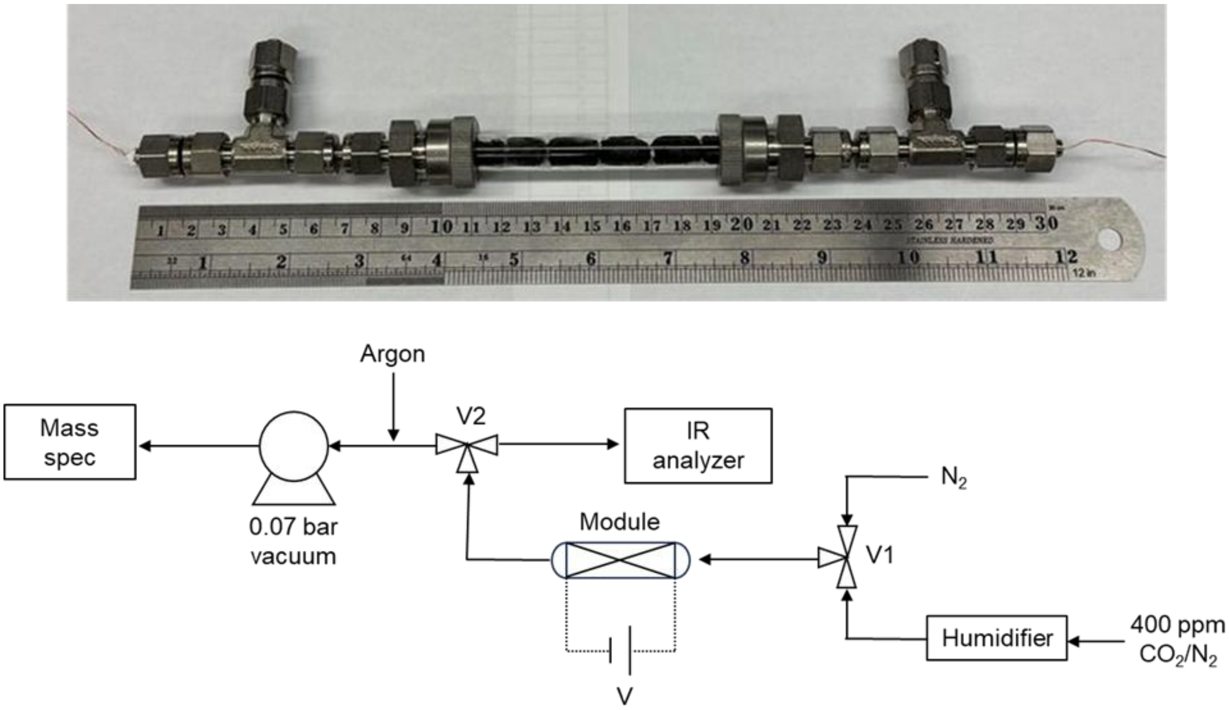

Figure S13. Prototype vacuum-assisted electrical thermal swing adsorption (V-ETSA) module (top) and a diagram for customized V-ETSA apparatus (bottom). PEI2.5(25k)-TAGO900 foams are electrically connected by conductive glue. During the actual V-ETSA process, the transparent glass tube was replaced with ½ inch stainless tube to prevent a vacuum leak.

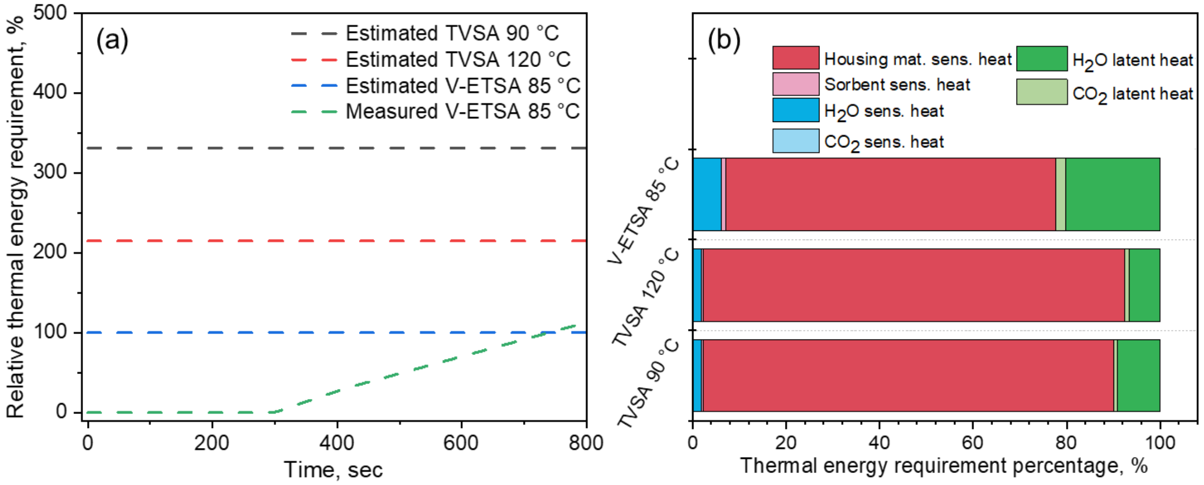

Figure S14. (a) Relative thermal energy requirements (normalized by the thermal energy requirements of the estimated V-ETSA process) of the studied desorption processes and (b) percentage contributions of energy components in the total thermal energy requirement. The thermal energy requirements for V-ETSA were measured via electrical input (i.e., voltage and current) during V-ETSA operation. The estimated thermal energy requirements were calculated by thermodynamic parameters and adsorption/desorption capacities from each process. The sensible heat contribution from the housing material (PTFE) is likely overestimated, as structural parameters such as the material thickness of the housing material were not optimized. The heat capacity (thermal mass) ratio of the housing material and adsorbent is 84. Nonetheless, this estimation enables a comparison of relative energy requirements within the identical desorption module.

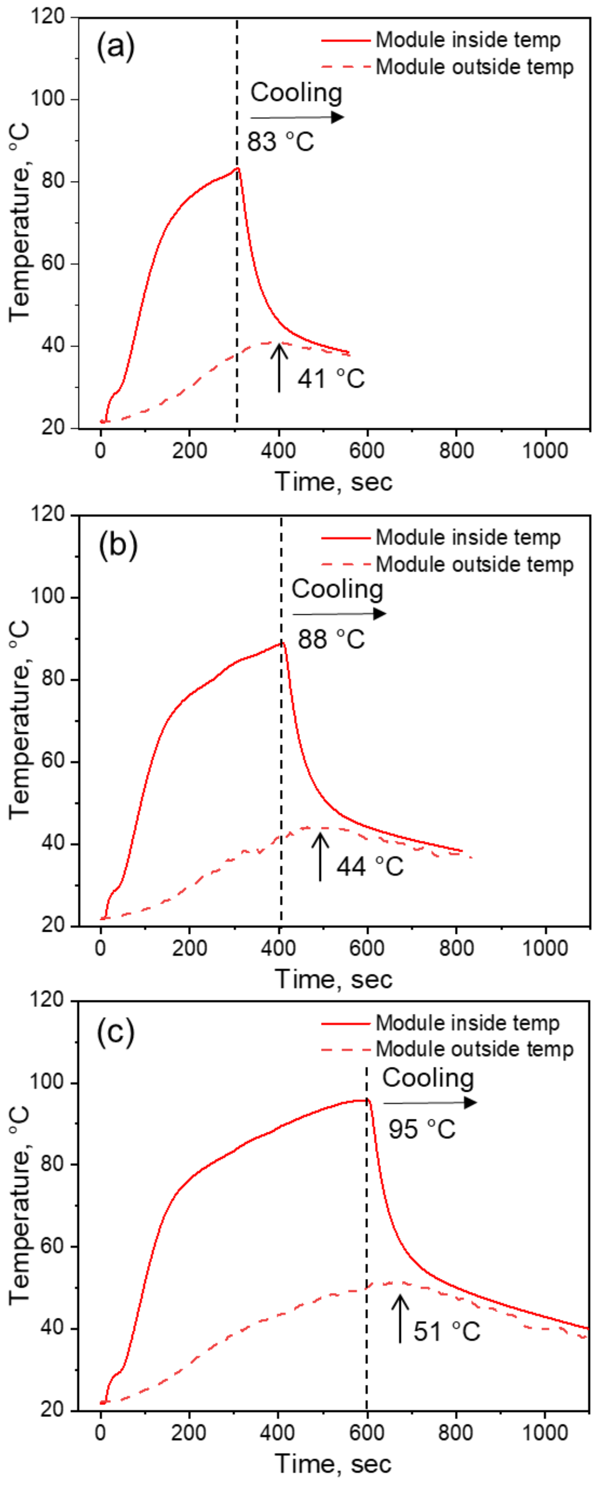

Figure S15. Comparison of cooling time after V-ETSA desorption of PEI2.5(25k)-TAGO900 at different module temperatures. If the target cool down temperature is 40 °C (before air is reintroduced), the cooling times for (a), (b), and (c) are 42, 268, and 414 sec, respectively. The rapid temperature decrease of the module inside temperature relative to the module outside temperature suggests that the cooling time is strongly affected by the module outside temperature.

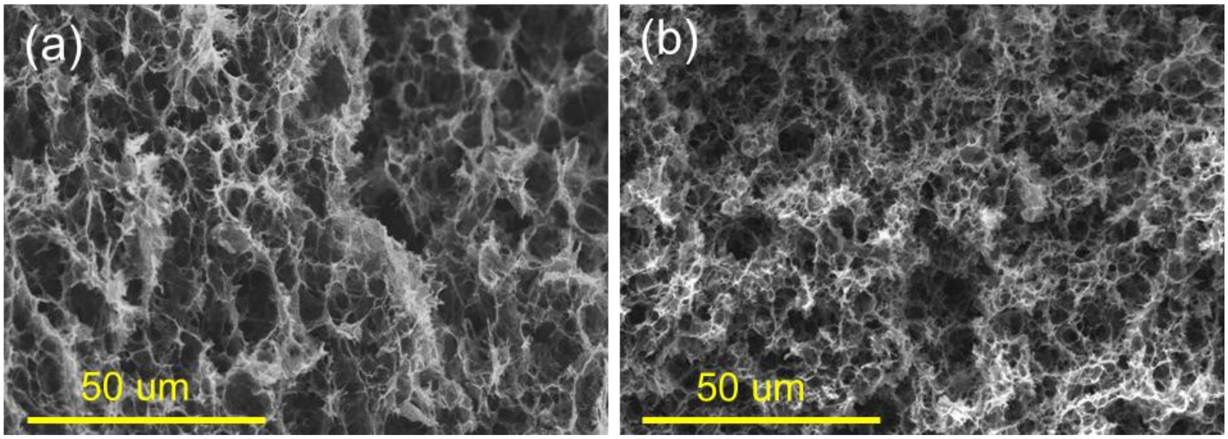

Figure S16. SEM images of PEI2.5(25k)-TAGO900 (a) before and (b) after 10 V-ETSA cycles under 0.07 mbar and ~85 °C.

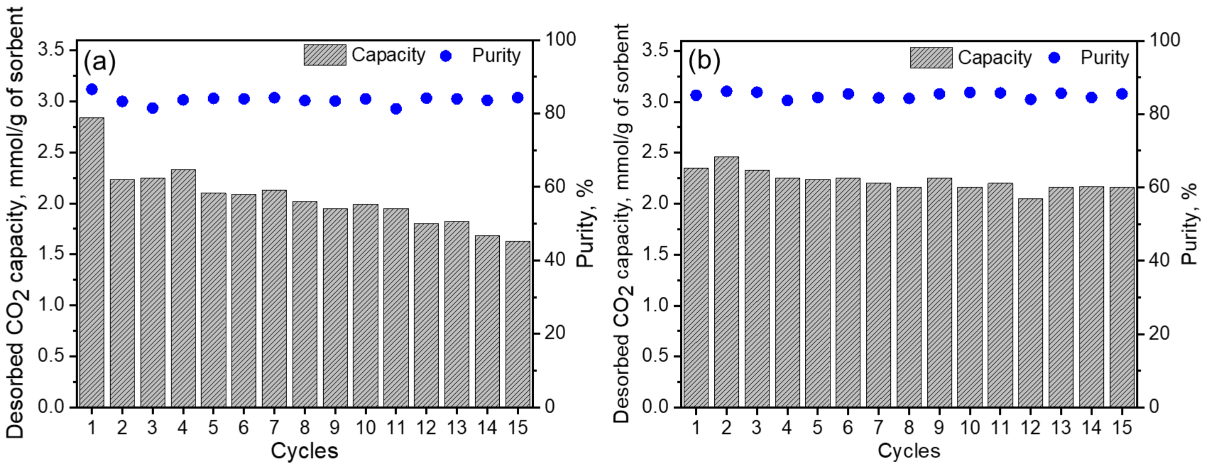

Figure S17. CO_2 desorption capacities and purities for (a) PEI5(800)-TAGO900 and (b) PEI5(25k)-TAGO900 samples during V-ETSA cycles. Desorption was performed under vacuum (0.07 bar) at 85 °C using a 4.5 V input. The degradation rate—defined as the loss in CO_2 adsorption capacity per cycle starting from the second cycle—was calculated to be 0.042 mmol/g/cycle for PEI5(800)-TAGO900 and 0.021 mmol/g/cycle for PEI5(25k)-TAGO900.

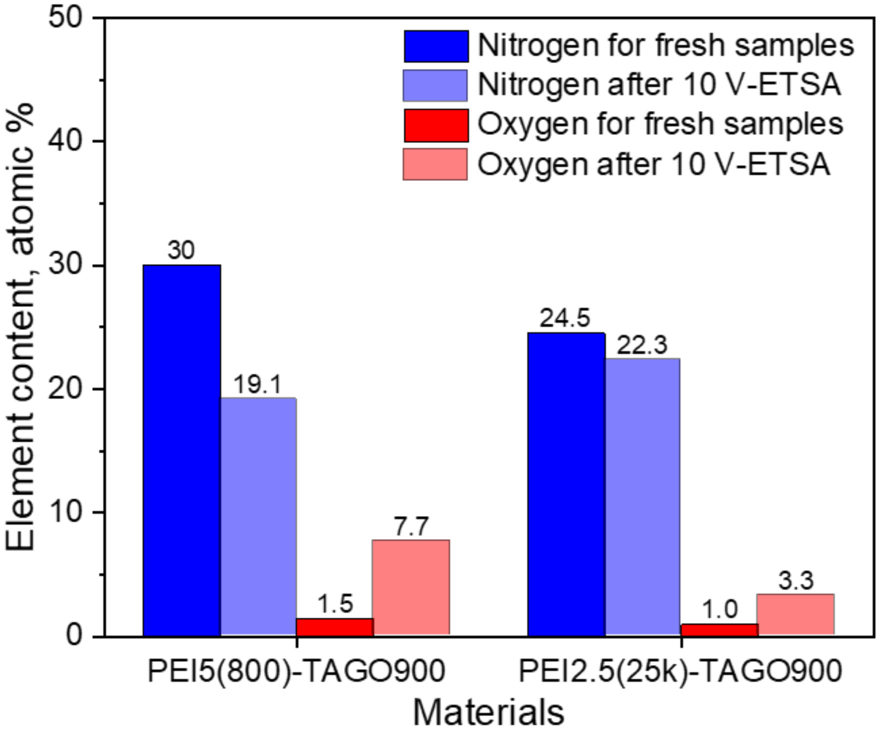

Figure S18. Nitrogen and oxygen contents of PEI5(800)-TAGO900 and PEI2.5(25k)-TAGO900 by XPS survey before and after 10 V-ETSA cycles under 0.07 bar and ~85 °C.

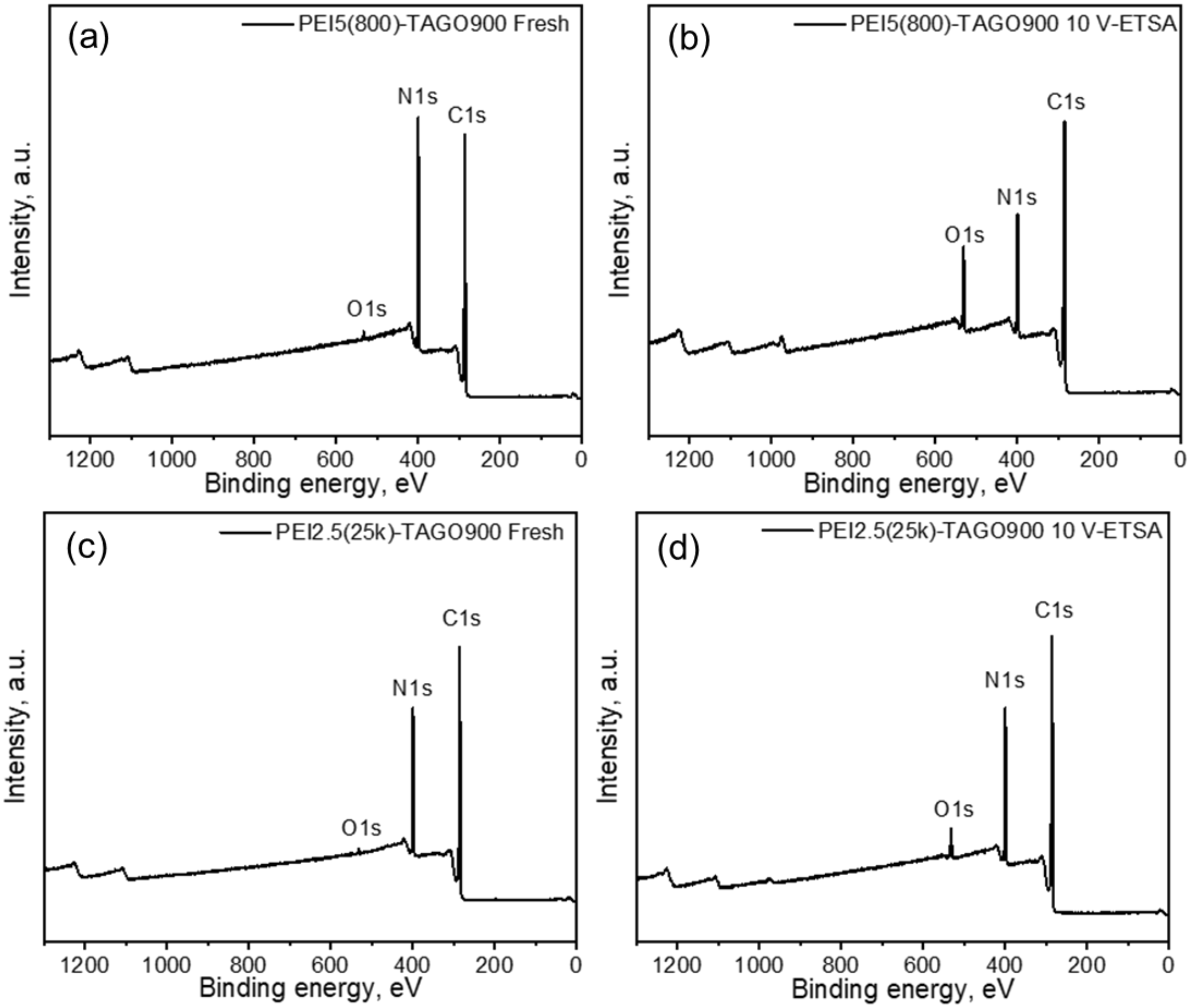

Figure S19. XPS survey spectra for PEI5(800)-TAGO900 and PEI2.5(25k)-TAGO900 (a and c) before and (b and d) after 10 V-ETSA cycles, respectively.

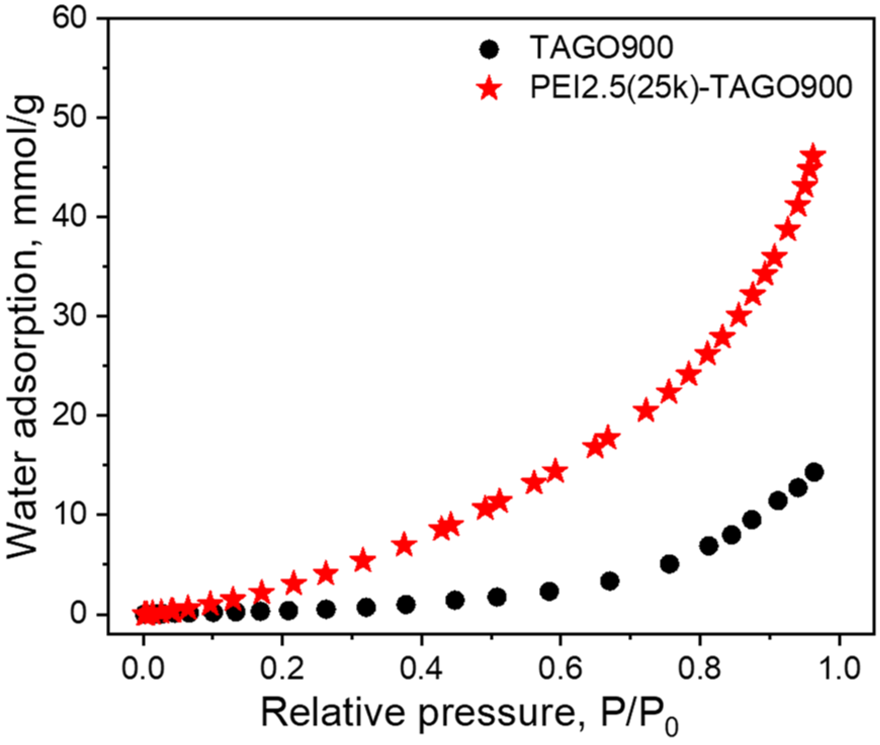

Figure S20. Water isotherms of TAGO900 and PEI2.5(25k)-TAGO900 at 25 °C. While water isotherms of TAGO900 indicate the hydrophobic nature of TAGO900, PEI functionalization increases the hydrophilicity of TAGO900 significantly.

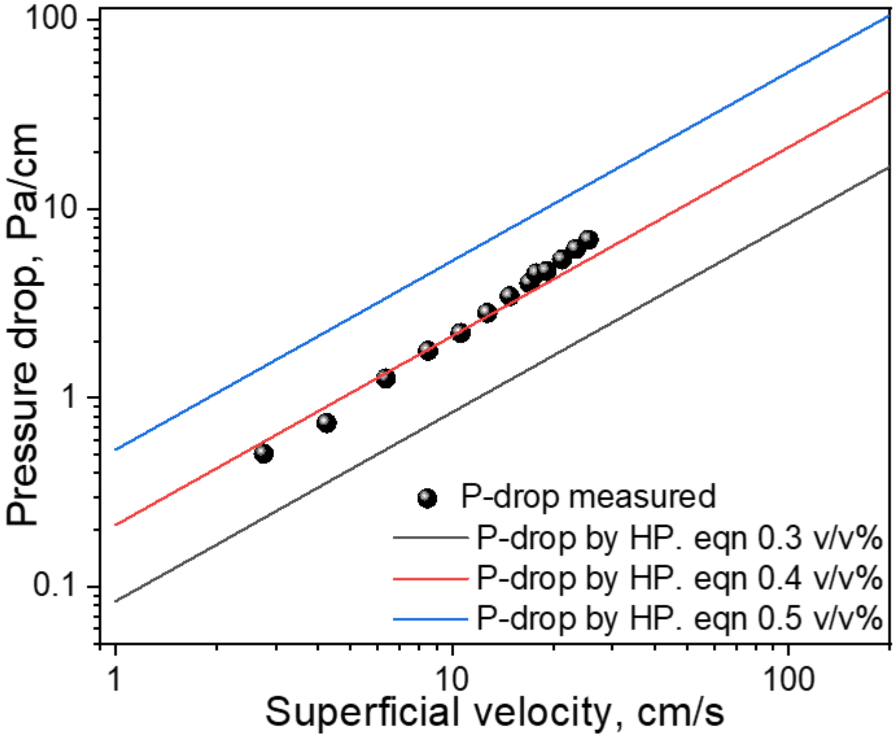

Figure S21. Pressure drops of PEI2.5(25k)-TAGO900 foams. The measured pressure drop (dots) follows the pressure drop estimated by the Hagen Poiseuille equation, which models the pressure drop of flow through cylinders.

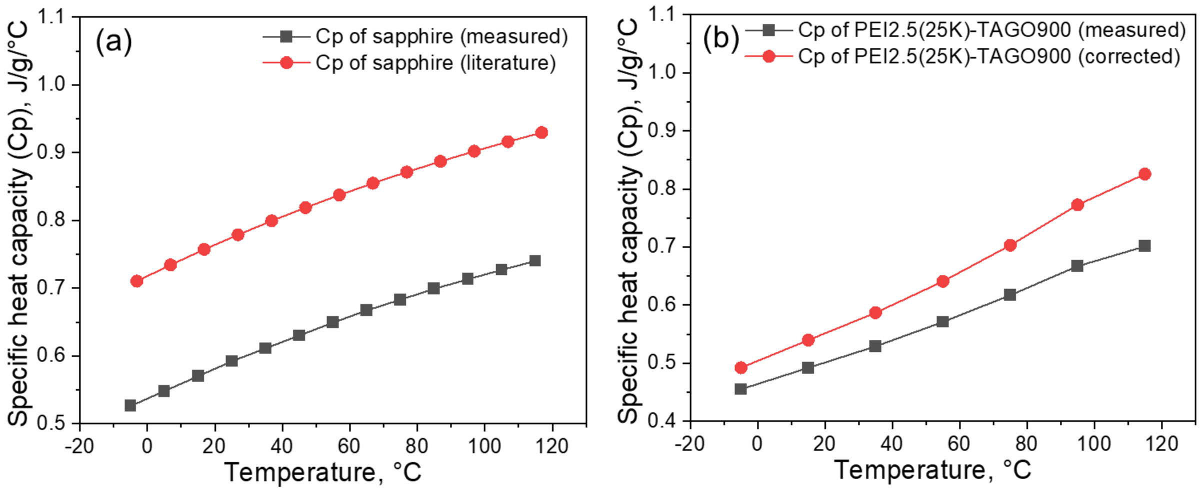

Figure S22. Specific heat capacity of (a) the standard sapphire sample and that of (b) PEI2.5(25k)-TAGO900. The specific heat capacity of PEI2.5(25k)-TAGO900 was corrected by applying a calibration factor derived from the ratio of the NIST-reported value for sapphire to the experimentally-measured value in this study.[5]

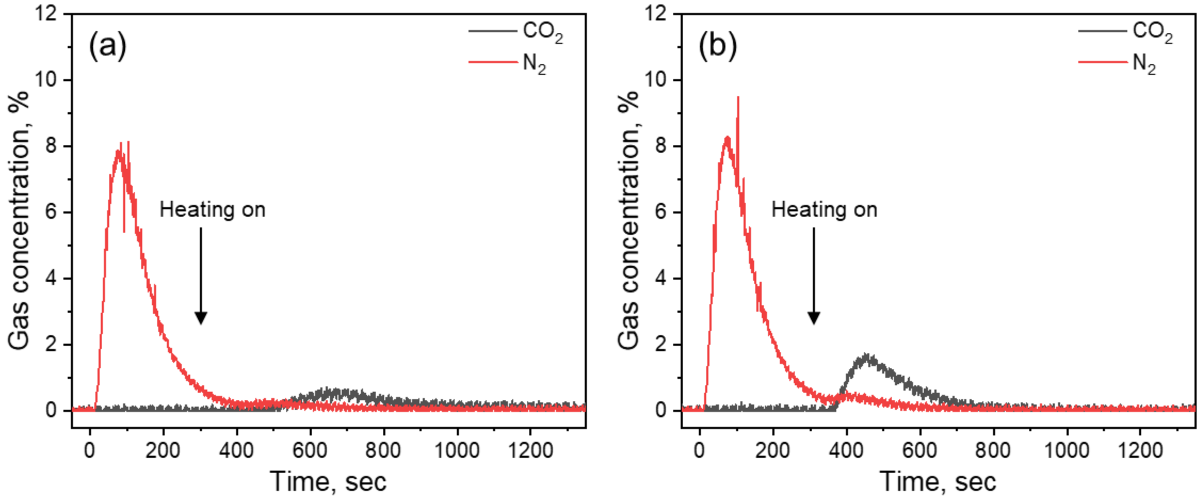

Figure S23. Desorbed gas concentration profiles for (a) TVSA at external temperature 90 °C and (b) V-ETSA at internal temperature 85 °C. V-ETSA showed sharper CO_2 desorption peak due to the faster heating and higher module internal temperature than TVSA system. Desorbed CO_2 purity was calculated by the desorbed CO_2 after heating is on divided by the sum of desorbed N_2 and CO_2 after heating is on. The desorbed CO_2 purity for TVSA and V-ETSA recorded 70 and 77%, respectively.

References

(1) Tuinstra, F.; Koenig, J. L. Raman spectrum of graphite. *The Journal of chemical physics* **1970**, *53* (3), 1126-1130.
(2) Chase, M. W. Data reported in NIST standard reference database 69, June 2005 release: NIST Chemistry WebBook. *J Phys Chem Ref Data Monograph* **1998**, *9*, 1.
(3) Furukawa, G. T.; McCoskey, R. E.; King, G. J. Calorimetric properties of polytetrafluoroethylene (Teflon). *J. Res. Natl. Bureau Stand.* **1952**, *49*, 273.
(4) Rim, G.; Priyadarshini, P.; Song, M.; Wang, Y.; Bai, A.; Realff, M. J.; Lively, R. P.; Jones, C. W. Support Pore Structure and Composition Strongly Influence the Direct Air Capture of CO2 on Supported Amines. *J. Am. Chem. Soc.* **2023**, *145* (13), 7190-7204. DOI: 10.1021/jacs.2c12707.
(5) Ditmars, D.; Ishihara, S.; Chang, S.; Bernstein, G.; West, E. Enthalpy and heat-capacity standard reference material: synthetic sapphire (α-Al2O3) from 10 to 2250 K. *Journal of Research of the National Bureau of Standards* **1982**, *87* (2), 159.
